# Identification of novel substrates of *Shigella* T3SA through analysis of its virulence plasmid-encoded secretome

**DOI:** 10.1371/journal.pone.0186920

**Published:** 2017-10-26

**Authors:** Laurie Pinaud, Mariana L. Ferrari, Robin Friedman, Nico Jehmlich, Martin von Bergen, Armelle Phalipon, Philippe J. Sansonetti, François-Xavier Campbell-Valois

**Affiliations:** 1 Unité de Pathogénie Microbienne Moléculaire, Institut Pasteur, Paris, France; 2 U1202, INSERM, Paris, France; 3 Laboratoire de Biologie Systémique & Centre de Bioinformatique, Biostatistique et Biologie Intégrative, Institut Pasteur, Paris, France; 4 Department of Molecular Systems Biology, Helmholtz-Centre for Environmental Research-UFZ, Leipzig, Germany; 5 Institute of Biochemistry, University of Leipzig, Faculty of Biosciences, Pharmacy and Psychology, Leipzig, Germany; 6 Chaire de Microbiologie et Maladies Infectieuses, Collège de France, Paris, France; 7 Department of Chemistry and Biomolecular Sciences, Centre for Chemical and Synthetic Biology, Faculty of Science, University of Ottawa, Ottawa, Ontario, Canada; 8 Department of Biochemistry, Microbiology and Immunology, Faculty of Medicine, University of Ottawa, Ottawa, Ontario, Canada; New York State Department of Health, UNITED STATES

## Abstract

Many human Gram-negative bacterial pathogens express a Type Three Secretion Apparatus (T3SA), including among the most notorious *Shigella spp*., *Salmonella enterica*, *Yersinia enterocolitica* and enteropathogenic *Escherichia coli* (EPEC). These bacteria express on their surface multiple copies of the T3SA that mediate the delivery into host cells of specific protein substrates critical to pathogenesis. *Shigella* spp. are Gram-negative bacterial pathogens responsible for human bacillary dysentery. The effector function of several *Shigella* T3SA substrates has largely been studied but their potential cellular targets are far from having been comprehensively delineated. In addition, it is likely that some T3SA substrates have escaped scrutiny as yet. Indeed, sequencing of the virulence plasmid of *Shigella flexneri* has revealed numerous open reading frames with unknown functions that could encode additional T3SA substrates. Taking advantage of label-free mass spectrometry detection of proteins secreted by a constitutively secreting strain of *S*. *flexneri*, we identified five novel substrates of the T3SA. We further confirmed their secretion through the T3SA and translocation into host cells using β-lactamase assays. The coding sequences of two of these novel T3SA substrates (Orf13 and Orf131a) have a guanine-cytosine content comparable to those of T3SA components and effectors. The three other T3SA substrates identified (Orf48, Orf86 and Orf176) have significant homology with antitoxin moieties of type II Toxin-Antitoxin systems usually implicated in the maintenance of low copy plasmids. While Orf13 and Orf131a might constitute new virulence effectors contributing to *S*. *flexneri* pathogenicity, potential roles for the translocation into host cells of antitoxins or antitoxin-like proteins during *Shigella* infection are discussed.

## Introduction

The virulence of several Gram-negative pathogenic bacteria, such as *Shigella spp*., *Salmonella enterica*, *Yersinia enterocolitica* and enteropathogenic *Escherichia coli* (EPEC), depends on expression of a Type Three Secretion System (T3SS). Its core component is the Type Three Secretion Apparatus (T3SA), which consists of a large insoluble protein assembly spanning the two bacterial membranes and extending away from the bacterial outer membrane through a fine hollow structure known as the needle. The T3SA allows the contact-mediated transfer of proteins also called effectors, from an attacking bacterium to an eukaryotic cell target (reviewed in [1]). Therefore, the delineation of the repertoire of T3SA substrates is a key step in understanding pathogenesis.

Genes in specific virulence-associated loci that encode proteins suspected to be T3SA substrates can be singled out from sequenced genomes and their translocation into host cells confirmed using various assays (reviewed in [2,3]). These assays rely on the fusion of putative substrates to an enzyme, such as the adenylate cyclase Cya or the β-lactamase TEM-1, whose activity can be quantified [4,5]. For instance, T3SA-mediated secretion of the *Chlamydia pneumoniae* proteins Inc identified by sequence homology has been demonstrated using the translocation assay based on the Cya reporter protein [6], and a machine learning classification algorithm identified twelve putative T3SA substrates of *P*. *aeruginosa*, among which two were found to be transferred into host cells using the β-lactamase translocation assay [7]. Another approach to identify T3SA substrates is based on mass spectrometry (MS). Stable isotope labelling by amino acid residues in cell culture (SILAC, [8]), and isobaric tagging for relative and absolute quantification (iTRAQ, [9]) have been used to detect translocated bacterial proteins in the lysate of infected host cells. For instance, SILAC identified six *C*. *rodentium* and two EPEC T3SA-substrates whose translocation within host cells was further confirmed using the β-lactamase assay [10,11], and iTRAQ enabled identification of twenty-six putative effectors of the Bsa T3SS of *Burkholderia pseudomallei* [12]. In contrast to protein labelling approaches, label-free MS techniques are easier to implement and have also proven useful in deciphering secretomes associated to pathogenic bacteria. For instance, label-free MS allowed identification of a novel secreted effector of *Vibrio cholerae* by comparing supernatants of bacterial strains expressing or not a Type Six Secretion Apparatus (T6SA) [13], which targets prokaryotic and/or eukaryotic cells [14,15].

By contrast, the T3SA secretome of *Shigella flexneri* has not been deeply scrutinised using proteomic approaches with only a single study focusing on the secretion hierarchy of known effectors being reported thus far [16]. In fact, only a few novel *Shigella* T3SA effectors have been discovered since the initial reports of the sequencing and annotation of the virulence plasmid [17,18]. Notable exceptions are OspI and IpaJ that have been suggested to be T3SA substrates when their effector functions were described [19,20]. In addition, five IpaH proteins encoded by seven genes on the bacterial chromosome are the only known T3SA substrates not encoded on the virulence plasmid [21]. Therefore, if a pool of unknown T3SA-substrates exists, a reasonable hypothesis is that it should be enriched among predicted proteins of unknown function encoded by open reading frames (ORFs) on the virulence plasmid.

*Shigella spp*. are Gram-negative enteropathogens that invade the large intestine of humans, resulting in an acute recto-colitis named bacillary dysentery. Each year, millions of children under five years of age are hospitalised worldwide due to severe diarrhoea, including dysentery, resulting in nearly 800,000 deaths mostly in low-income countries [22]. *Shigella* T3SA is essential for the pathogenesis of this facultative intracellular pathogen. Indeed, the T3SA and its substrates mediate invasion of host cells and lysis of bacteria-containing vacuole allowing spreading of the infection to neighbouring cells (reviewed in [23]). Colonisation of the large intestine mucosa is also facilitated by T3SA effectors that modulate the host immune response, which would otherwise circumscribe the infection (reviewed in [24]). In addition to the comprehensive annotation of *S*. *flexneri* virulence plasmid [17,18], study of its T3SA-dependent secretome is facilitated by bacterial genetics groundwork that has identified useful mutant strains. For example, the *ipaD* strain displays a constitutively active T3SA resulting in release of T3SA substrates in large amounts in the bacterial growth medium, while the WT strain releases only small amount of T3SA substrates under the same conditions [25]. In contrast, the *mxiD* strain does not build functional T3SA [26], hence proteins released in its growth medium are secreted in a T3SA-independent manner (e.g. through other secretion systems, non-canonical secretion pathway such as outer membrane vesicles or passive release from dead bacteria).

Herein, we have exploited label-free MS combined with the extensive knowledge of the genetic circuitry regulating activity of the *Shigella* T3SA and β-lactamase assays to study the T3SA secretome encoded on the virulence plasmid of *S*. *flexneri*. Using this approach, we have confirmed the secretion of most known or suspected T3SA substrates and identified five novel substrates that may constitute additional molecular weapons in the *Shigella* arsenal.

## Materials and methods

### Bacterial strains, cloning reagents and plasmids

Experiments were performed with the wild-type *S*. *flexneri* 5a strain M90T-Sm harbouring a streptomycin resistance mutation [27] and/or its derivative mutant strains *ipaD* [28] and *mxiD* [26]. *Shigella* strains were grown on trypticase soy broth (TSB) or trypticase soy agar in conditions indicated under the relevant sub-headings below.

The plasmids used for expression of bacterial proteins fused to the β-lactamase TEM-1 (referred to as Orf-bla chimeric proteins) are derived from pSU2718 (GenBank: M64731) with the following alterations: (i) modification of the multiple cloning site; (ii) upstream of the lac promoter, addition of the transcription terminator of *trpA* by mutagenesis PCR (resulting plasmid pSU2.1tt) and (iii) downstream of the lac promoter *via* KpnI/XbaI restriction sites, addition of the sequence coding for the mature β-lactamase TEM-1 isolated from pUC18, with 5’ insertion of a sequence coding for a six amino acid linker (resulting plasmid pSU2.1tt-bla). The sequences coding for the *Shigella* Orfs of interest were extracted from M90T pWR100 virulence plasmid DNA (using the annotations from [17]) and inserted in frame upstream of the linker *via* EcoRI/SmaI restriction sites (resulting plasmids pSU2.1tt-Orf-bla). A “consensus” Shine and Dalgarno (SD) sequence isolated from pQE-60 (see [29]) was part of the forward primer used for PCR amplification of each sequence inserted into pSU2.1tt and pSU2.1tt-bla. For each Orf, exchange of this “consensus” SD for the “endogenous” SD (endSD) sequence was performed either by mutagenesis PCR or by repeating extraction of the Orf of interest together with the 30 to 40 nucleotides upstream of its start codon in pWR100 sequence. Where indicated, mutagenesis PCR on the resulting pSU2.1tt-endSD-Orf-bla plasmids was performed to exchange the β-lactamase coding sequence for the c-Myc coding sequence (resulting plasmids pSU2.1tt-endSD-Orf-myc). The primers and plasmids used are listed in S1 and S2 Tables. NucleoSpin Plasmid miniprep kit (Macherey-Nagel), PCR clean up and gel extraction kits (Macherey-Nagel), PureLink HiPure midiprep kit (Invitrogen) were used for DNA preparation. Restriction enzymes, T4 DNA ligase, T4 polynucleotide kinase, Taq DNA polymerase and Phusion polymerase were purchased from ThermoFisher Scientific. Chemically competent *Escherichia coli* DH5α bacteria (Invitrogen) were used for all cloning steps and were grown at 37°C in 2xYeast Tryptone medium, supplemented with 30 μg/mL chloramphenicol when transformed with Orf-bla/myc-expressing plasmids.

*Shigella* strains were transformed with plasmids expressing Orf-bla or Orf-myc by electroporation and isolated on TSB agar supplemented with 0.01% Congo red and 10 μg/mL chloramphenicol after overnight incubation at 37°C. When indicated, WT *Shigella* strains expressing Orf-bla chimeric proteins were transformed with pUC18Δz-DsRed, a plasmid allowing constitutive expression of the fluorescent DsRed.T3 protein [30]. This plasmid was obtained by exchanging the insert of the pUC18Δz-TSAR plasmid for the DsRed coding sequence under the control of the *rpsM* promoter from the pTSAR1.3 plasmid, *via* SapI/HindIII restriction sites (both pUC18Δz-TSAR and pTSAR1.3 plasmids are described in [29], the former corresponding to the pFX4 plasmid with the coding sequence conferring resistance to zeocin instead of ampicillin). *Shigella* Orf-bla strains transformed with pUC18Δz-DsRed were isolated on TSB agar supplemented with 0.01% Congo red, 10 μg/mL chloramphenicol and 300 μg/mL zeocin after overnight incubation at 37°C.

### Mass spectrometry data collection

*mxiD* and *ipaD* strains were grown overnight at 30°C and sub-cultured 1/50 in TSB at 37°C for 6 hours. Sub-cultures were spun at 3,220 g, 4°C for 15 min and supernatants filtered with 0.45 μm syringe filters. Proteins contained in the filtered supernatants were precipitated overnight at 4°C with 10% (v/v) trichloroacetic acid. Protein pellets from centrifugation at 9,400 g, 4°C for 15 min were recovered in 400 μL Laemmli buffer 1X (Biorad), and pH re-equilibrated with a 2M sodium hydroxide solution. Proteins present in the lysates were separated using polyacrylamide gel electrophoresis (SDS-PAGE) followed by Coomassie G-250 staining (Merck). Each gel lane corresponding to a unique experimental condition was cut into a single piece, then de-stained, dehydrated and treated with trypsin (Promega) overnight at 37°C. The resulting peptides were desalted using C_18_ ZipTip column (Merck Millipore) and subsequently dissolved in 0.1% formic acid prior to liquid chromatography mass spectrometry analysis (LC-MS/MS). MS was performed on an Orbitrap Fusion MS (Thermo Fisher Scientific, Waltham, MA, USA) with a TriVersa NanoMate (Advion, Ltd., Harlow, UK) source in LC chip coupling mode. The peptide lysates were separated on a UHPLC system (Ultimate 3000, Dionex/Thermo Fisher Scientific, Idstein, Germany). 5 μL samples were first loaded for 5 min on the pre-column (μ-precolumn, Acclaim PepMap, 75 μm inner diameter, 2 cm, C18, Thermo Scientific) at 4% mobile phase B (80% acetonitrile in nanopure water with 0.08% formic acid), 96% mobile phase A (nanopure water with 0.1% formic acid), then eluted from the analytical column (PepMap Acclaim C18 LC Column, 25 cm, 3 μm particle size, Thermo Scientific) over a 120 min linear gradient of mobile phase B (4–55% B). The mass spectrometer was set on top speed with a cycle time of 3 s, using the Orbitrap analyser for MS and MS/MS scans with higher energy collision dissociation (HCD) fragmentation at normalised collision energy of 28%. MS scans were measured at a resolution of 120,000 in the scan range of 400–1,600 *m/z*. MS ion count target was set to 4×10^5^ at an injection time of 60 ms. Ions for MS/MS scans were isolated in the quadrupole with an isolation window of 2 Daltons (Da) and were measured with a resolution of 15,000 in the scan range of 350–1,400 *m/z* in the Orbitrap. The dynamic exclusion duration was set to 30 s with a 10-ppm tolerance around the selected precursor and its isotopes. Automatic gain control target was set to 5×10^4^ with an injection time of 120 ms.

### Proteome data analysis

Proteome Discoverer (v1.4, Thermo Scientific) was used for protein identification and the acquired MS/MS spectra were searched with SEQUEST HT algorithm against the Uniprot reviewed database of *Shigella* pWR100 proteins (database of 265 protein-coding sequences, containing pWR100 protein annotations from NCBI [17] and the *S*. *flexneri* chromosomal IpaH sequences, combined with the sequences of 150 common contaminants). Enzyme specificity was selected to trypsin with up to two missed cleavages allowed using 10-ppm peptide ion tolerance and 0.05 Da MS/MS tolerances. Oxidation (methionine) and carbamylation (lysine and arginine) were selected as variable modifications and carbamidomethylation (cysteine) as a static modification. Only peptides with a false discovery rate (FDR) <0.01 calculated by Percolator [31] and peptide rank = 1 were considered as identified. All peptides matching more than one protein were removed, because they could not be used to distinguish between the secretion of homologs (in particular peptides matching the constant carboxy-terminal domain of IpaH proteins). The only exceptions to this rule were OspE1/OspE2, IpaH1.4/IpaH2.5, IpaH1/IpaH6 and IpaH4/IpaH5 for which this filter would have removed nearly all peptides identified, as these proteins are almost identical (>98% identity at the amino acid level). Therefore, these protein pairs were treated as one entity and appear as a single entry in the MS data analysis. The abundance of each detected protein was obtained by averaging peak intensities of up to 3 of its most abundant peptides in each replicate [32,33]; abundance values obtained from at least three independent replicates were then used to derive the average abundance value. Proteins identified in the *ipaD* sample were sorted into hit tiers based on the following criteria: Tier 2 hits had either at least 1.5-fold higher abundance for peptides also identified in the *mxiD* sample (*i*.*e*. shared peptides), or no peptides identified in the *mxiD* sample for at least two identified in the *ipaD* sample; Tier 1 hits had either no peptides identified in the *mxiD* sample for one identified in the *ipaD* sample, or 1.5-fold more peptides detected in the *ipaD* sample with a relative abundance lower than 1.5-fold; Tier 0 hits were the proteins not falling into the previous categories.

### Congo red induction, bacterial lysates and immunoblotting

Relevant *Shigella* strains were grown overnight at 30°C with the appropriate antibiotics and sub-cultured 1/50 in TSB at 37°C without antibiotics. When indicated, T3SA secretion of WT strains was induced *in vitro* after the bacterial sub-culture had reached an optical density at 600 nm (OD_600_) of 0.1, through addition of 30 μg/mL of the T3SA inducer Congo Red (CR) for 4 hours. This protocol was essentially performed as in [34,35] except that a lower concentration of CR was used, as in [36]. Sub-cultures were spun at 9,000 g for 1 min and equivalent amounts of bacteria were resuspended in Laemmli buffer for pellet fractions (Biorad). For supernatant fractions, supernatants were filtered with 0.22 μm syringe filters and secreted proteins precipitated on ice for 30 min using 10% (v/v) trichloroacetic acid. Protein pellets resulting from spinning at 16,000 g for 30 min were resuspended in Laemmli buffer. Boiled lysates were analysed by SDS-PAGE and the following antibodies were used to detect proteins of interest: mouse anti-β-lactamase (Abcam ab12251 or Novus Biologicals NB120-12251; 1/1,000), rabbit anti-c-Myc (Santa Cruz sc789; 1/750), rabbit anti-RecA (Abcam ab63797; 1/500), rabbit anti-IpaH ([37,38]; 1/5,000), rabbit anti-Spa15 ([39,40]; 1/20,000), anti-mouse-HRP (GE Healthcare NXA931; 1/10,000), anti-rabbit-HRP (Cell Signalling 7074S; 1/7,000). Membranes were imaged using film detection or Amersham Imager 600 (GE Healthcare). When indicated, quantification of band intensity was performed using Image Studio^TM^ Lite software (LI-COR Biosciences).

### Secretion assay

Relevant *Shigella* strains were grown overnight at 30°C with the appropriate antibiotics and sub-cultured 1/50 in TSB at 37°C for 4 hours without antibiotics. β-lactamase activity was measured by incubating 20 μL of the colorimetric β-lactamase substrate nitrocefin (500 μg/mL stock, EMD Millipore) with 100 μL of different dilutions of bacterial supernatants (after spinning sub-cultures at 9,000 g for 1 min) or cleared sonicated sub-cultures (after spinning sonicated sub-cultures at 10,600 g for 1 min). Sonication of sub-cultures was performed with a UP50H sonicator (Hielscher Ultrasound Technology) by continuous sonication over 30 seconds at 50% amplitude. β-lactamase-mediated hydrolysis of the nitrocefin substrate was assessed by measuring absorbance at 486 nm (A_486_) over time using a plate reader (Infinite M200 Pro; TECAN). β-lactamase activity was calculated in equivalent Miller units (e.M.u.) and averaged over successive time points within the linear part of the curve: Activity [e.M.u.] = 1,000 x (A_486_ [sample]—A_486_ [TSB]) / (time(min) x volume(mL) x OD_600_[subculture] x sample dilution).

### Host cell infection for translocation assays

Jurkat T cells (clone E6-1, ATCC TIB-152) and HeLa cells were respectively cultured in RPMI1640 and DMEM 1 g/L glucose (Gibco), supplemented with 10% heat-inactivated foetal calf serum (HI-FCS) at 37°C with 5% CO_2_. HeLa cells were seeded the day before the experiment at 0.5x10^5^ cells per cm^2^. Prior to infection, cells were washed and put back in the incubator in assay medium (i.e. DMEM containing 2.5 mM probenecid (VWR), which is an anion transport inhibitor facilitating the retention of CCF2-AM within loaded cells) supplemented with 2 μM CCF2-AM (ThermoFisher Scientific). Jurkat T cells were seeded on the day of the experiment in 96-well plates round-bottom at 3-5x10^5^ cells per well in assay medium (i.e. RPMI1640 containing 2.5 mM probenecid) supplemented with 1 μM CCF2-AM. Relevant bacterial strains grown overnight at 30°C with the appropriate antibiotics were sub-cultured 1/50 in TSB at 37°C without antibiotics until early exponential phase was reached. Sub-cultures were adjusted in the appropriate assay medium to the indicated multiplicity of infection (MOI). CCF2-AM-loaded Jurkat or HeLa cells were infected by centrifugation of bacteria at a MOI of 50 onto the cells at 300 g, 37°C for 10 min. Alternatively, bacterial sub-cultures were coated with 10 μg/mL poly-L-lysine (Sigma) in PBS for 10 min, prior to infection of CCF2-AM-loaded HeLa cells for 15 min at room temperature at a MOI of 10. Infection was subsequently allowed to proceed for indicated times at 37°C with 5% CO_2_. When indicated, host cells were incubated in assay medium supplemented with 50 μg/mL gentamycin to kill extracellular bacteria. Sample analyses of the translocation assay for both cell types are described in the next section.

### Translocation assay data acquisition and analysis

Infected HeLa cells were washed three times in PBS containing 2.5 mM probenecid. Cells were then switched to the imaging medium composed of DMEM without Phenol Red (Gibco) containing 2.5 mM probenecid and 50 μg/mL gentamycin. Acquisitions were performed using an Axio Observer.Z1 microscope (Zeiss) equipped with a swept field confocal Opterra system (Bruker) and an Evolve 512 Delta EMCCD camera (Photometrics), using a 40x PlanAPOCHROMAT oil immersion/1.4 NA objective (Zeiss). Fluorescence images were sequentially acquired as follows: (i) excitation at 405 nm and detection with a 460/50 filter (Blue channel, cleaved CCF2-AM); (ii) excitation at 405 nm and detection with a 531/50 filter (FRET channel, uncleaved CCF2-AM); (iii) excitation at 488 nm and detection with a 535/50 filter (Green channel, total CCF2-AM); (iv) excitation at 561 nm and detection with a 630/75 filter (Red channel, DsRed bacteria). Confocal Z-stacks (15 Z-slices of 0.5 μm) were acquired using a 70 μm slit, with an exposition time of 50 ms. Post-acquisition image analysis and quantification were performed with Fiji 2.0.0 software [41]: regions of interest (ROI) were defined for invaded cells loaded with CCF2-AM (based on Red and Green channels) and for each ROI the total mean fluorescence of the 15 Z-slices in the Blue and FRET channels were used to calculate the cleaved/uncleaved CCF2-AM fluorescent ratio (Blue/FRET channels).

Infected Jurkat cells were washed in PBS containing 2.5 mM probenecid. They were then stained with the Live/Dead near-infrared dead cell stain kit (ThermoFisher Scientific) following the supplier guidelines except that 2.5 mM probenecid was maintained during all steps. Cells were finally resuspended in the flow cytometry buffer composed of ice-cold PBS containing 2.5 mM probenecid and 0.1% BSA. Samples were acquired on a BD FACSCanto II flow cytometer (BD Bioscience). Single cells were gated based on forward and side scatters and dead cells were excluded based on excitation with the 633 nm laser coupled to 750/60 nm emission filter. Cleaved and uncleaved CCF2-AM were detected within live cells with the 405 nm excitation laser and 450/50 and 510/50 nm emission filters, respectively. Data were analysed using Flow Jo 10.0.8 software.

### Plaque assay

The *Shigella* single-mutant strains used in plaque assays were the non-spreading strain *icsA* [42] and *orf86*, *orf13*, *orf131a* and *orf176* [43]. The tetracycline cassette flanked by flippase recognition target (FRT) sites present within each of the latter strains was removed upon transformation with the pCP20 plasmid that encodes the flippase recombinase (FLP), as described in [44]. Plaque assays were performed as described previously [45], using Caco-2/TC7 cells cultured to confluency for three weeks in DMEM 1 g/L glucose supplemented with non-essential amino acids, glutamine (all from Gibco) and 20% HI-FCS at 37°C with 10% CO_2_. Following 2 hours of infection with 10^6^ bacteria at 37°C with 10% CO_2_, an overlay of 0.5% agarose containing 50 μg/ml gentamycin in culture medium was applied to extensively washed infected monolayers. Three days later, ethanol fixation and Giemsa R solution (RAL Diagnostics) staining allowed for enumeration of plaques and measurement of their area using Fiji 2.0.0 software [41].

### Data presentation and statistical analysis

Prism 6.0 (GraphPad Software, Inc.) was used for graphs and statistical analyses. Unpaired two-tailed Student’s t-test was used to compare two conditions unless indicated otherwise. Significant statistical differences are indicated by asterisks: *p≤0.05; **p≤0.01; ***p≤0.001; ****p≤0.0001. Error bars represent standard deviation to the mean. Illustrator CS5 software (Adobe) was used for assembling figures.

## Results

**Fig 1:**
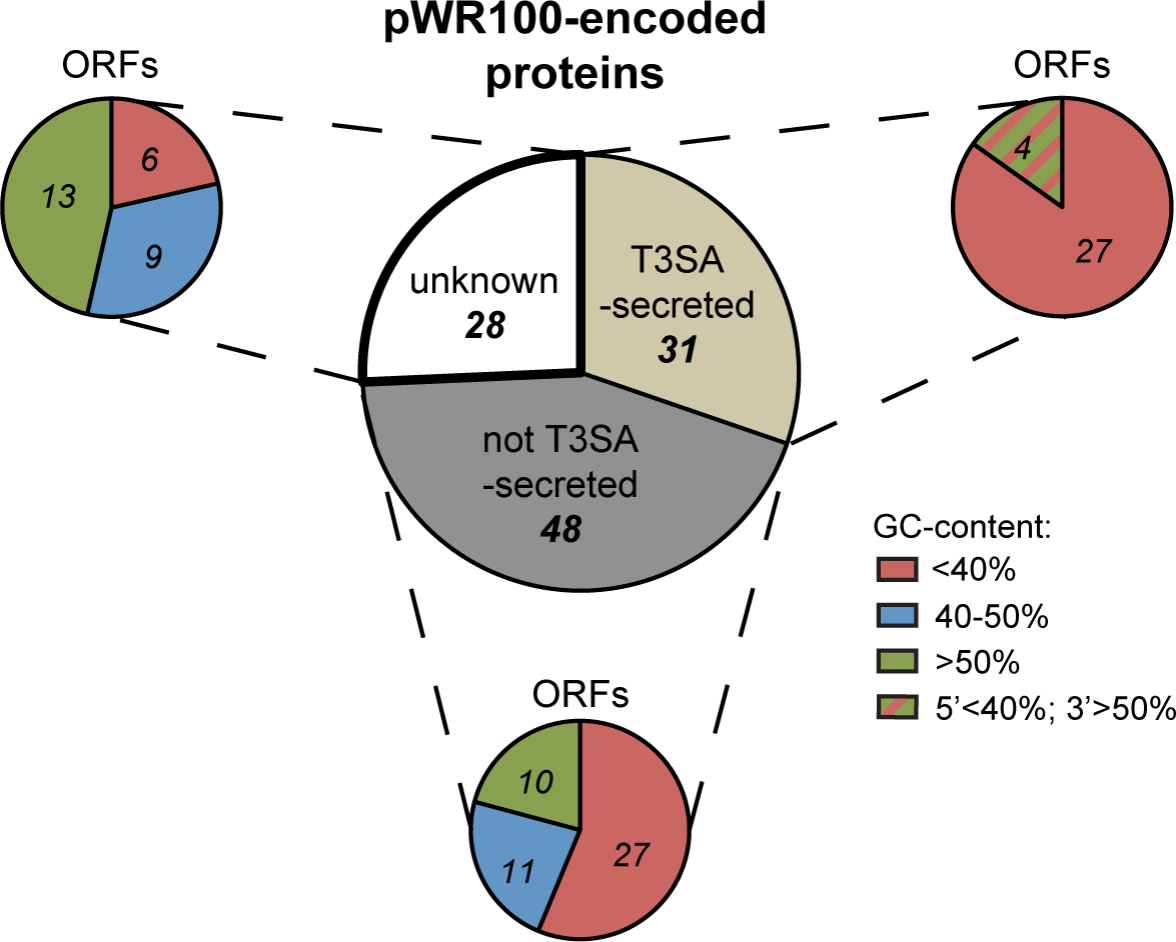
pWR100-encoded proteins. Proteins encoded on pWR100 virulence plasmid classified based on their T3SA-dependent secretion and GC-content of the corresponding genes.

To define the pool of potential T3SA substrates, we listed the basic properties of genes annotated on the virulence plasmid pWR100 from *S*. *flexneri* strain M90T [17]. This indicated that the virulence plasmid encodes 31 known or suspected T3SA substrates (i.e. proteins secreted by the T3SA), 48 cytoplasmic, periplasmic or membrane proteins, and 28 annotated proteins with unknown localisation (Fig 1). T3SA substrates encoded by duplicate genes, such as OspE1/OspE2 and IpaH1.4/IpaH2.5 that share 99% and 98% identity, respectively, were considered as single T3SA-secreted proteins [17]. Based on their guanine-cytosine content (GC-content), genes encoded by the virulence plasmid seem to have arisen from a minimum of three independent acquisition events [17]. Importantly, the T3SA and most of its substrates are encoded by genes with a GC-content lower than 40% (Fig 1). The only exception is the IpaH family whose members are encoded by chimeric genes displaying T3SA-like GC-content (< 40%) in 5’ and a higher GC-content in 3’ (> 50%) (Fig 1). In addition to the four IpaH proteins encoded on the virulence plasmid, seven genes on the bacterial chromosome encode five other members of the IpaH family [21]. As chromosomally-encoded IpaH1/IpaH6 and IpaH4/IpaH5 are 99% identical, each pair was considered a unique T3SA-secreted protein.

To investigate whether pWR100-encoded proteins with unknown localisation could be part of the T3SA secretome of *S*. *flexneri*, we designed a strategy relying on: (i) label-free quantitative MS to identify secreted bacterial proteins; (ii) statistical analysis of MS data based on the number and abundance of peptides identified in the supernatants of strains displaying high versus no T3SA-dependent secretion (*i*.*e*. *ipaD* versus *mxiD*). This approach allowed us to identify T3SA substrate candidates with a high level of confidence. T3SA-mediated secretion into bacterial growth medium and translocation into human cell lines of the candidates were then tested using β-lactamase assays (for a general overview of the study design, see S1 Fig).

### Identification by mass spectrometry of proteins secreted by *Shigella* T3SA

Proteins found in the culture medium of *ipaD* and *mxiD S*. *flexneri* strains were recovered by trichloroacetic acid precipitation and analysed by MS. These MS data are retrievable using the dataset identifier PXD006086 from the ProteomeXchange Consortium *via* the PRIDE partner repository [46]. Detected peptides were identified by mining the pWR100 annotated proteins database supplemented with the five chromosomally-encoded IpaH proteins [17]. For each of the three biological replicates, an identified protein was classified as a hit tier 2, 1 or 0, based on the quantification of abundance and number of matching peptides detected in *ipaD* versus *mxiD* supernatants (tier 2 hits are the proteins identified as T3SA substrates with the highest confidence). Proteins classified as tier 2 hits in at least two biological replicates were categorised as high confidence predictions while intermediate confidence predictions were tier 2 or 1 hits in at least two biological replicates. Lastly, proteins classified as tier 2 hits in only one biological replicate were categorised as low confidence predictions.

**Fig 2:**
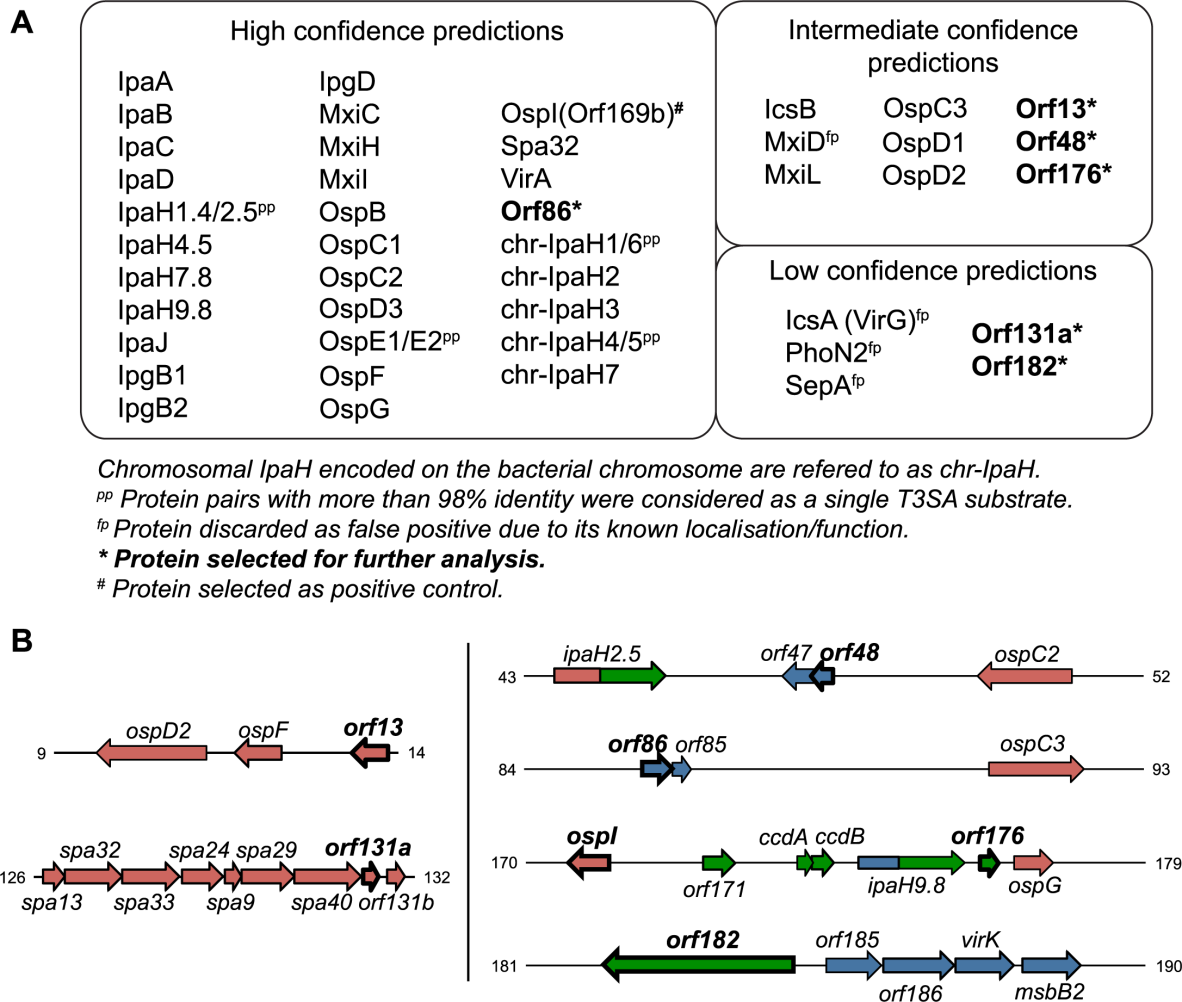
Mass spectrometry results. (**A**) Group of proteins predicted to be T3SA substrates with low, medium and high confidence using the MS approach described in the text. Novel putative substrates and control substrate OspI selected for further analysis are highlighted in bold. (**B**) Genetic mapping of the ORFs selected for further investigation. Colours refer to the GC-content of the genes: red <40%; blue 40–50%; green >50%. Numbers refer to the coordinates on pWR100 sequence in kilobases (accession number AL391753).

This analysis of the MS results classified the chromosomally-encoded IpaH proteins and 30 out of 31 previously known or highly suspected T3SA substrates encoded on pWR100 as high or intermediate confidence predictions (Fig 2A and S3 Table). Only OspZ, the product of *orf212*, was not detected in the secreted fraction. OspZ shares homology with the enteropathogenic *E*. *coli* effector NleE and was previously found to be secreted by *S*. *flexneri 2a* strain 2457T [47]. However, expression of *ospZ* in the *S*. *flexneri* 5a strain M90T used herein has been reported to be among the lowest of all genes located on the virulence plasmid [48], which might explain why it was undetected in the secreted fraction. On the other hand, four proteins of unknown function and localisation, namely Orf86, Orf13, Orf48 and Orf176, were predicted at high or intermediate confidence to be T3SA substrates. By contrast, identification of the outer membrane protein MxiD as an intermediate confidence prediction is an experimental artefact. Indeed, most of the *mxiD* coding sequence is replaced with an antibiotic resistance cassette, precluding detection of several MxiD-matching peptides in the *mxiD* strain, but not in the *ipaD* strain (see [26] for description of the *mxiD* mutant). Besides, three proteins identified as low confidence predictions were discarded on the basis of their known cellular localisation: IcsA (VirG) and SepA are auto-transporters [49,50] and PhoN2 is a periplasmic enzyme [51]. Nevertheless, two proteins of unknown function and localisation named Orf131a and Orf182 were predicted at low confidence to be T3SA substrates. Furthermore, the MS analysis validated the T3SA-dependent secretion of OspI (Orf169b), whose effector function was recently uncovered [19]. Because mRNA expression of *ospI* was comparable to that of *orf13* [48], and their peptide abundance in *ipaD* supernatant were similar (S3 Table), OspI was selected as a positive control for following experiments. Therefore, the proteins selected for further analysis were the novel putative T3SA substrates (i.e. Orf86, Orf48, Orf176, Orf13, Orf131a and Orf182) and the T3SA effector OspI.

### Features of mass spectrometry hits

The six T3SA substrate candidates are encoded by *orf13*, *orf48*, *orf86*, *orf176*, *orf131a* and *orf182*. Overall these genes are well conserved with more than 98% sequence identity with their orthologs in the four *Shigella spp*. (*i*.*e*. *S*. *flexneri*, *boydii*, *sonnei* and *dysenteriae*). The only exceptions were *orf48* and *orf86* for which no orthologs were found in *S*. *sonnei*. As represented in Fig 2B, *orf13* and *orf131a* exhibit a GC-content below 40%, which is a hallmark of known T3SA substrates [17]. *orf131a*, also known as *SPA-ORF10*, is located at the end of the *mxi-spa* locus. Its protein product named uncharacterized 9.1 kDa protein in spaS 3'region (www.uniprot.org), which we called Orf131a for the sake of simplicity (as in [17]), exhibits 27% sequence identity with *E*. *coli* YmgB/AriR, which has been implicated in biofilm formation repression and acid-resistance [52]. *orf13* is located 1031 bp upstream of the gene coding for the known T3SA substrate OspF [17] and its protein product Orf13 has no identified conserved domain or characterised homolog. Two of the MS hits encoded by genes with a higher GC-content, namely *orf48* and *orf86* (44.9% and 42.9%, respectively), share a domain predicted to interact with DNA (Clusters of Orthologous Group 4453, COG4453) and are related to the antitoxin component of type II toxin-antitoxin (TA) systems [53] (*orf48* and *orf86* being referred to as *0063* and *0112* in [53], based on another virulence plasmid annotation [18]). Plasmid-encoded TA systems are classically described as post-segregation killing systems ensuring the maintenance of low-copy plasmids by inducing death of plasmid-free daughter cells (reviewed in [54]). Recently, McVicker and colleagues reported that Orf48/0063, but not Orf86/0112, was part of a functional TA system contributing to the maintenance of *S*. *flexneri* large virulence plasmid under certain growth conditions [53]. Another MS hit, Orf176, encoded by a gene with a GC-content of 50.6%, displays a conserved domain related to predicted transcriptional regulators (COG3905) and shares 93% identity with the antitoxin-like YacA plasmid stabilisation protein in *E*. *coli* (Orf176 is referred to as YacA in [53]). A sequence coding for a protein similar to its cognate toxin YacB is found downstream of the *orf176* gene on pWR100. However, presence of two supplementary nucleotides creates a premature stop codon, therefore probably inactivating the toxin of this putative *yacA/yacB* homolog [53]. Noteworthy, *orf176* is intriguingly located between *ipaH9*.*8* and *ospG*, which both encode T3SA substrates [17]. Lastly, *orf182* displays a high GC-content (50.9%) and its protein product has a molecular weight higher than typical T3SA substrates. Orf182 harbours an ankyrin repeat domain that suggests a potential role in protein-protein interactions.

### Validation of T3SA-dependent secretion of the novel T3SA substrates using a β-lactamase secretion assay

**Fig 3:**
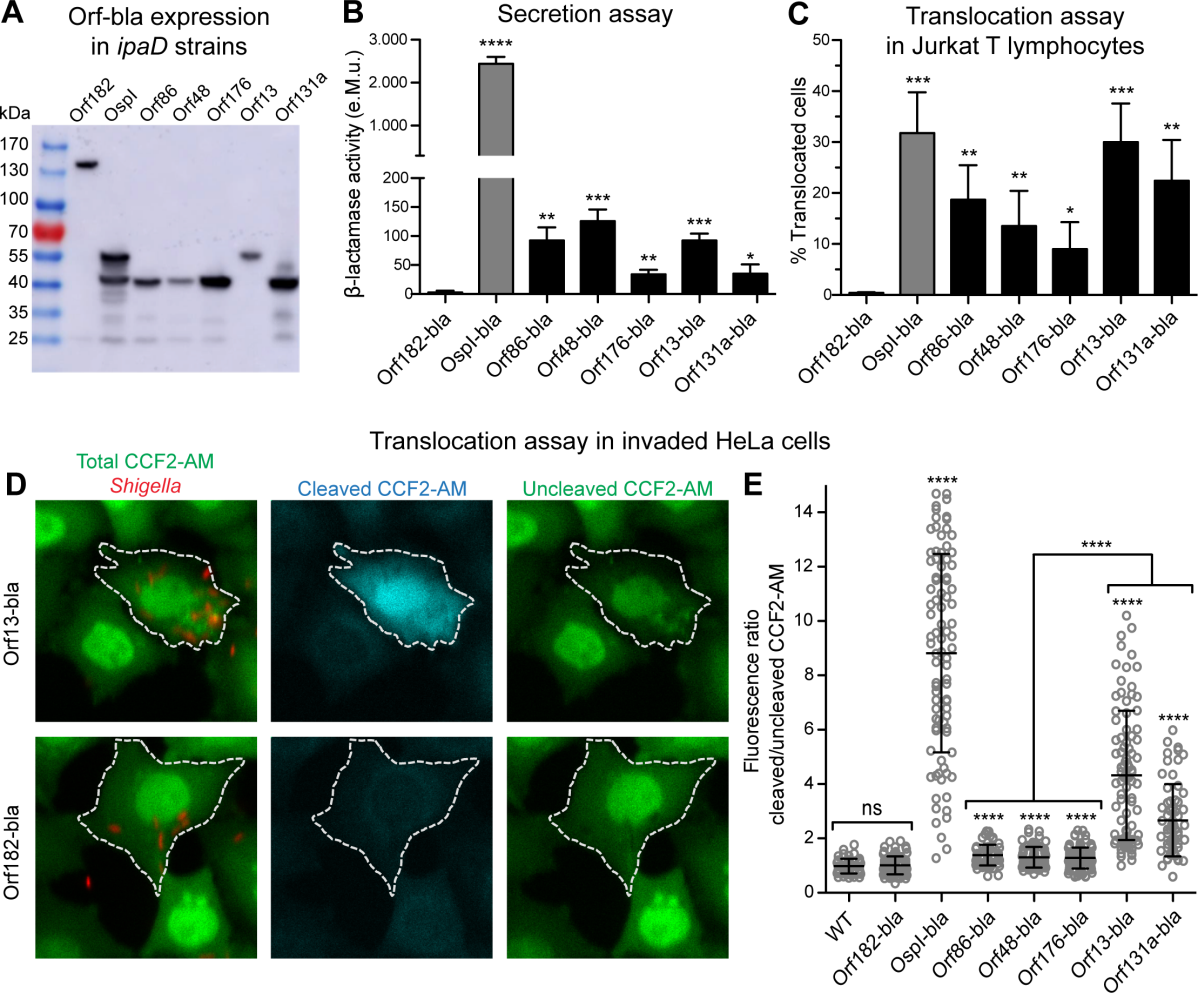
Experimental confirmation of secretion and translocation of MS hits. (**A**) *ipaD Shigella* lysates were analysed by immunoblotting with anti-β-lactamase antibody. Load equivalent to a bacterial culture optical density at 600 nm (OD_600_) of 0.2 for each lane. Chimeric proteins tested and their expected molecular weight (kiloDaltons, kDa): Orf182-bla (140), OspI-bla (53), Orf86-bla (40), Orf48-bla (41), Orf176-bla (40), Orf13-bla (51), Orf131a-bla (38). (**B**) Supernatants of *ipaD* strains expressing the Orf-bla chimeric proteins were incubated with nitrocefin. Enzymatic activity was calculated based on measurement of absorbance at 486 nm (A_486_). e.M.u.: equivalent Miller unit. Data are from 3 independent experiments. *p<0.05; **p<0.01; ***p<0.001; ****p<0.0001 (unpaired two-tailed Student’s t-test compared to Orf182-bla). (**C**) CCF2-AM-loaded Jurkat T lymphocytes were infected with WT strains expressing Orf-bla chimeric proteins for 1 hour. Data are from 4 independent experiments. Translocated cells were detected by flow cytometry. *p<0.05; **p<0.01; ***p<0.001 (unpaired two-tailed Student’s t-test compared to Orf182-bla). (**D-E**) CCF2-AM-loaded HeLa cells were infected with WT-DsRed strains expressing Orf-bla chimeric proteins for 1 hour, followed by 1 hour of incubation with gentamycin. (**D**) Representative images of cells infected with WT *Shigella* strains expressing the indicated chimeric proteins, showing total CCF2-AM (Green channel), cleaved CCF2-AM (Blue channel) and uncleaved CCF2-AM (FRET channel). Dashed lines denote cells invaded by DsRed bacteria (Red channel) that were used for fluorescence quantification. (**E**) CCF2-AM cleavage was quantified within invaded cells based on fluorescence intensity of cleaved over uncleaved CCF2-AM within the defined regions of interest. Data are from 3 independent experiments, representing 50 to 100 invaded cells analysed per condition. ****p<0.0001 (unpaired two-tailed Welch’s t-test compared to Orf182-bla unless depicted otherwise).

To test by an independent approach whether the T3SA substrate candidates identified by MS were indeed secreted by *S*. *flexneri* T3SA, we generated plasmids enabling expression of chimeric proteins composed of an amino-terminal T3SA substrate candidate and a carboxy-terminal β-lactamase (bla) moieties. The coding sequence for MS hits and the selected positive control were cloned individually into a low copy expression plasmid containing the coding sequence for the mature β-lactamase TEM-1 (*i*.*e*. lacking the signal peptide required for periplasm localization). For each β-lactamase construct, we chose to initiate translation of the chimeric proteins from the “endogenous” Shine and Dalgarno (SD) sequence encoded by the 30 to 40 nucleotides upstream of each of the selected ORFs in pWR100. Indeed we observed that this allowed an optimal expression when compared to the use of a constant “consensus” SD sequence (S2 Fig). The “endogenous” SD-based plasmids were introduced into *ipaD* strains and expression of the chimeric proteins was confirmed in bacterial lysates by immunoblotting using an antibody raised against the β-lactamase (Fig 3A). Most Orfs-bla yielded a single prominent band of the expected molecular weight. OspI-bla yielded a band corresponding to the predicted molecular weight of 53 kDa and a smaller band of approximately 45 kDa discussed below. Secretion of the Orf-bla proteins was assessed using an *in vitro* secretion assay: supernatants from the *ipaD* strains were incubated with nitrocefin, a colorimetric substrate of β-lactamase [55]. Absorbance at 486 nm was measured to assess hydrolysis of the substrate and thus quantify β-lactamase activity. Despite Orf182-bla being well expressed by *ipaD* bacteria (Fig 3A), very faint β-lactamase activity was detected in the supernatant of that strain (Fig 3B). This was not due to misfolding of the β-lactamase when fused to Orf182 because upon sonication-induced release of the cytosol, Orf182-bla processed nitrocefin similarly to OspI-bla and Orf48-bla (S3A Fig). Moreover, Orf182 was not secreted when fused to a smaller protein such as Myc while OspI-myc was (S3B Fig), ruling out the possibility that T3SA-mediated secretion of Orf182 was prevented due to the large size of the resulting β-lactamase chimeric protein. Altogether, this demonstrated that the low-confidence MS prediction Orf182 was a false positive from the MS analysis. Therefore, Orf182-bla was used as a non-secreted control in the following experiments. Of note, the additional lower molecular weight species in the strains expressing OspI-bla (Fig 3A) or OspI-myc (S3B Fig) displayed a similar size difference compared to the highest molecular weight species. This suggested that the lower molecular weight species in both lysates were due to an intrinsic feature of OspI rather than to the carboxy-terminal tag used. These lower molecular weight species were not secreted (S3B Fig), indicating that they did not contribute to the β-lactamase activity detected in *ipaD* supernatant (Fig 3B). Since the tag detected by immunoblotting is located in the carboxy-terminus of the chimeric proteins, these lower molecular species are likely amino-terminal truncations that might be produced by proteolytic digestion or from translation initiation at an internal start site, similarly to what was recently shown for an amino-terminal peptide of OspD1-bla [56].

In comparison to Orf182-bla, significantly more enzymatic activity was detected in the supernatants of *ipaD* strains expressing the positive control OspI-bla or the five other Orf-bla chimeric proteins (Fig 3B). Differences in enzymatic activity between these *ipaD* strains could not be explained by variations in Orf-bla expression (Fig 3A) because no linear correlation was found between the β-lactamase activity detected in supernatants and the Orf-bla protein expression (R^2^ = 0.005; see S4A Fig for protein quantification). Furthermore, the β-lactamase activity in *ipaD* supernatants was not due to bacterial lysis as the cytosolic protein RecA was not detected in secreted fractions (S3C Fig). As suggested by the MS analysis, Orf-bla secretion was T3SA-dependent because no enzymatic activity was detected in supernatants of T3SA-deficient *mxiD* strains expressing the chimeric proteins (S3D and S3E Fig). In addition, we assessed Orf-bla secretion by the WT strains through induction of the T3SA activity by the dye Congo Red (CR). Briefly, bacterial cultures at an optical density of 0.1 were supplemented or not with CR and allowed to grow for 4 hours until the late exponential-early stationary phase was reached. Because CR absorbs in the same wavelength as the cleaved nitrocefin, we assessed Orf-bla secretion by immunoblotting with the anti-β-lactamase antibody (S3F Fig). In the absence of CR, all Orf-bla but Orf182-bla were detected in WT supernatants. This is in agreement with a previous report indicating that in bacterial cultures grown beyond the late exponential phase, T3SA substrates such as the translocators IpaB and IpaC were significantly secreted in the absence of CR [57]. In the presence of CR, all Orf-bla but the Orf182-bla were present in the supernatants and in most cases more abundant compared to the basal condition. Taken together, these results support the notion that Orf86, Orf48, Orf176, Orf13 and Orf131a are secreted in a T3SA-dependent manner by *S*. *flexneri*.

### Validation of T3SA-mediated translocation of the novel T3SA substrates into human cells using a β-lactamase translocation assay

Translocation of the novel T3SA substrates into host cells was then investigated by infecting human cell lines with WT *S*. *flexneri* strains expressing the different chimeric proteins (S5A Fig). As expected, no β-lactamase activity could be detected in the supernatants of these WT strains upon *in vitro* growth, except for OspI-bla that was secreted to a lower extent than in the *ipaD* strain (S5B Fig). Delivery of β-lactamase chimeric proteins into the host cell cytoplasm upon infection was assessed using an assay based on the cell-permeable fluorescence resonance energy transfer (FRET) β-lactamase substrate CCF2-AM [58]. Presence of the β-lactamase within the cytoplasm of cells loaded with this fluorescent dye results in the cleavage of CCF2-AM, which yields an easily detectable green-to-blue shift in the emission fluorescence wavelength [5,55,59]. We first investigated Orf-bla translocation into T lymphocytes by taking advantage of the accuracy of flow cytometry to measure translocation into the Jurkat T lymphocyte cell line, similarly to what was previously described using IpgD-bla-expressing WT bacteria [59]. The fluorescence intensity of cleaved versus uncleaved CCF2-AM within each cell was measured. Uninfected cells were used as the negative control to set the gate defining translocated host cells (S5C Fig). Using this gating strategy, we obtained the percentage of translocated Jurkat T cells, which was significantly higher for all T3SA substrate candidates compared to the non-translocated Orf182-bla (Fig 3C). The absence of cleaved CCF2-AM signal in Jurkat T cells infected with *mxiD* strains expressing a subset of Orf-bla confirmed that their translocation was T3SA-dependent (S5D and S5E Fig).

Next, we investigated whether translocation of the five novel substrates could be detected as well in human epithelial cells. To do this, we infected CCF2-AM-loaded HeLa cells with WT-DsRed *Shigella* strains expressing the different chimeric proteins. Infected cells were imaged using confocal microscopy (Fig 3D) and the fluorescence ratio between cleaved and uncleaved CCF2-AM was calculated for several invaded cells in each condition (Fig 3E). Cells invaded by Orf182-bla-expressing *Shigella* displayed a fluorescence ratio similar to that of cells invaded with WT bacteria devoid of Orf-bla (Fig 3E). The positive control OspI-bla and all the Orf-bla chimeric proteins induced cleavage of the CCF2-AM probe within invaded HeLa cells, as shown by the significantly higher fluorescence ratio compared to Orf182-bla-infected cells (Fig 3E). Intracellular bacterial lysis was excluded as a possible explanation for CCF2-AM cleavage as cells invaded with the WT strain expressing Orf182-bla did not display any significant substrate cleavage as compared to WT-invaded cells, while the β-lactamase was shown to be functional when fused to Orf182 (S3A Fig). In agreement with the *ipaD* secretion assay (Fig 3B), OspI-bla was more translocated into HeLa cells than the other Orf-bla chimeric proteins (Fig 3E). In contrast to what was previously described for the β-lactamase secretion assay (Fig 3B), translocation of Orf13-bla and Orf131a-bla into HeLa cells was significantly higher than that of the antitoxin-based Orf-bla (Fig 3E). These differences could not be explained by the variations in Orf-bla expression by WT *Shigella* (S5A Fig) as no significant linear correlation was found with CCF2-AM fluorescence ratio (R^2^ = 0.24; see S4B Fig for protein quantification). Overall, the translocation assay into HeLa cells confirmed the results obtained with the Jurkat human T cell line, further asserting that Orf13, Orf131a, Orf86, Orf48 and Orf176 are T3SA substrates.

To test whether these T3SA substrates contributed to *Shigella* capacity to multiply and disseminate within a cell monolayer, we used a plaque formation assay in a human colonic epithelial cell line. Both the number and the average size of plaques formed by relevant single-mutant strains were indistinguishable from the WT (S6 Fig). This suggested that Orf86, Orf176, Orf13 and Orf131a are not involved in the molecular events necessary for cellular spread in tissue culture cells. The role of Orf48 could not be assessed because as *orf48*-*47* encode a fully functional TA system [53], the *orf48* mutant strain displaying a complete deletion of the sequence coding for the antitoxin moiety of this TA module could not be obtained.

### Investigation of the role of protein size in the β-lactamase secretion and translocation assays, using known antitoxins and chaperones

**Fig 4:**
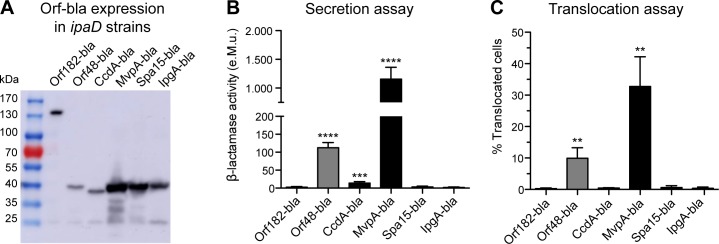
Investigation of secretion and translocation of antitoxins and chaperones. (**A**) *ipaD Shigella* lysates were analysed by immunoblotting with anti-β-lactamase antibody. Load equivalent to a bacterial culture OD_600_ of 0.2 for each lane. Chimeric proteins tested and their expected molecular weight (kDa): Orf182-bla (140), Orf48-bla (41), CcdA-bla (38), MvpA-bla (38), Spa15-bla (44), IpgA-bla (44). (**B**) Supernatants of *ipaD* strains expressing the Orf-bla chimeric proteins were incubated with nitrocefin. Enzymatic activity was calculated based on measurement of A_486nm_. e.M.u.: equivalent Miller unit. Data are from 4 independent experiments. (**C**) CCF2-AM-loaded Jurkat T cells were infected with WT strains for 1 hour. Translocated cells were detected by flow cytometry. Data are from 3 independent experiments. (**B**-**C**) **p<0.01; ***p<0.001; ****p<0.0001 (unpaired two-tailed Student’s t-test compared to Orf182-bla).

Strikingly, the novel T3SA substrates are relatively small proteins with four out of the five weighing approximately 10 kDa. To test whether these proteins were translocated because of their small size, we assessed the secretion of the 15 kDa Spa15 and IpgA T3SA effector chaperones [39,60]. In addition, since three newly identified T3SA substrates are related to antitoxin components of TA systems, we also investigated whether CcdA and MvpA (8 and 9 kDa, respectively), the antitoxins of two additional TA systems of pWR100 [61,62], could also be T3SA substrates. Indeed, such small proteins may have gone undetected by the mass spectrometer particularly if a small fraction of the total amount is secreted. The same strategy as for the confirmation of the T3SA-dependent secretion of the MS hits was employed to assess secretion into *ipaD* supernatants and translocation into host cells upon infection with WT strains. Orf182-bla was used as a negative control and the antitoxin-containing Orf48-bla was selected as a positive control displaying a molecular weight similar to that of proteins tested in this second set. Expression of this second set of Orf-bla chimeric proteins was confirmed within bacterial lysates (Figs 4A and S7A), secretion into *ipaD* and WT supernatants was assessed using the nitrocefin secretion assay (Figs 4B and S7B) and translocation into host cells investigated upon infection of Jurkat T cells (Fig 4C). The Orf-bla chimeric proteins based on the chaperones Spa15 and IpgA were neither significantly secreted into *ipaD* supernatants (Fig 4B) nor translocated into host cells upon infection with WT strains (Fig 4C). This was not due to inactivity of the β-lactamase when fused to these proteins because nitrocefin hydrolysis was detected upon sonication-induced bacterial lysis (S7C Fig). Interference of the β-lactamase with the secretion of Spa15 and IpgA is unlikely because secretion of Spa15-Myc and IpgA-Myc by *ipaD* strains was also undetected (S7D Fig). Interestingly, a study previously reported T3SA-mediated secretion of Spa15 at late time points [36]. A hypothesis explaining this discrepancy may be that the expression of chimeric proteins in our assays blocks secretion of Spa15. This was however invalidated by the failure to detect endogenous Spa15 in the supernatants of *ipaD* or CR-induced WT strains (S7E and S7F Fig). Since we studied a serotype 5a strain (M90T) while Spa15 secretion was previously reported in a serotype 2a strain (2457T) [36], an alternative hypothesis is that the behaviour of Spa15 might vary from strain to strain. Overall, the results obtained with T3SA effector chaperones support that the secretion and translocation of the novel T3SA-substrates is not an experimental artefact due to their small size.

Similarly to the chimeric proteins based on the antitoxin or antitoxin-like proteins Orf86, Orf48 and Orf176, MvpA-bla was secreted by *ipaD* bacteria in a T3SA-dependent manner (Figs 4B and S7G) and translocated into host cells by WT strains (Fig 4C). On the contrary, CcdA-bla was faintly secreted by the *ipaD* strain when compared to Orf182-bla (Fig 4B). However, the β-lactamase activity detected in *ipaD* supernatant was not different from that in *mxiD* supernatant (S7G Fig) and CcdA-bla was not translocated into host cells by WT bacteria (Fig 4C). We eliminated the possibility that failure to detect CcdA-bla translocation was due to its low expression as it was comparable to that of Orf48-bla (Fig 4A), which is efficiently translocated into host cells (Fig 4C). Therefore, CcdA was not further considered a T3SA substrate.

## Discussion

In this study, we have identified five novel T3SA substrates encoded on *S*. *flexneri* virulence plasmid pWR100: Orf13, Orf131a, Orf176, Orf86 and Orf48. The endogenous proteins were detected in higher amounts in the supernatant of the hyper-secretive *ipaD* strain in comparison to the T3SA-deficient *mxiD* strain. Their identification relied on the annotation of the virulence plasmid and the availability of genetic mutants, allowing us to devise a simple *in vitro* system to recover the secreted fraction of proteins from bacterial broth and to identify T3SA substrates using an easy to implement label-free quantitative MS approach. Transfer of the five newly identified T3SA substrates from the bacterial cytoplasm into the bacterial broth or into host cells in a T3SA-dependent manner was further confirmed with β-lactamase assays. Elucidation of the specific roles of these T3SA substrates in the pathogenesis of *Shigella* infection will be the focus of future studies.

β-lactamase assays revealed some discrepancies between the relative secretion and translocation of the novel T3SA substrates. While according to the nitrocefin secretion assay the novel T3SA substrates behaved quite similarly, the antitoxins (Orf48, Orf86 and Orf176)-bla generated significantly lower signal than Orf13-bla and Orf131a-bla in translocation assays. One can hypothesise that upon infection of eukaryotic cells, antitoxin-based proteins could be less favoured T3SA substrates compared to proteins encoded by genes with a GC-content similar to that of the T3SS. Another factor that might be playing a role in this phenomenon is the short half-life of antitoxins stemming from their sensitivity to proteolytic digestion [54]. Since proteases are probably present in low amounts in the bacterial growth medium, the Orf-bla would be relatively protected from protease digestion in this environment. In contrast, exposure to more abundant proteases in the host cytoplasm would reduce to a greater extent the concentration of labile antitoxins (Orf48, Orf86 and Orf176)-bla than that of Orf13-bla or Orf131a-bla. As shown for GFP protein fusions in mammalian cells, the stability of the GFP moiety of a chimeric protein can be reduced by fusion to a partner with a short half-life [63]. We hypothesise that the same basic principle applies to the β-lactamase in Orf-bla, hence leading to lower relative signal in the translocation versus secretion assays for antitoxin-based Orf-bla.

The similarity in GC-content (30–40%) between *orf13*, *orf131a* and genes associated with the T3SS is consistent with a shared evolutionary origin. However, the protein products Orf13 and Orf131a have very poor homology with known effectors and harbour no or very low sequence identity with conserved protein domains, hence complicating the elaboration of a hypothesis about their function. The genes encoding the three other novel T3SA substrates seem to have been acquired independently from the T3SS as they display a higher GC-content: *orf48* (44.9%), *orf86* (42.5%) and *orf176* (50.4%). The corresponding proteins are related to antitoxin components of type II TA systems. *orf48* and the partially overlapping downstream *orf47* were recently found to encode for a functional TA involved in the stability of the virulence plasmid under different growth conditions (*orf48-orf47* are referred to as *0063*–*0062* in [53]). Downstream of *orf86*, *orf85* is predicted to encode a GCN5-related N-acetyltransferase (GNAT) similar to Orf47. However, overexpression of Orf85 had no impact on bacterial growth, suggesting that Orf86-85 was not acting as a functional TA system in the conditions tested (*orf86-orf85* are referred to as *0112*–*0111* in [53]). Lastly, Orf176 seems to be an orphan antitoxin, as its downstream cognate toxin is probably not expressed due to the introduction of two supplementary nucleotides resulting in a premature stop codon (*orf176* is referred to as *yacA* in [53]). We also identified the better-known antitoxin MvpA, but not CcdA, as a T3SA substrate using β-lactamase assays. One cannot exclude that secretion of the antitoxins in these assays is higher than normal because of increased amount of free antitoxin-bla due to the disruption of the endogenous ratio of toxin to antitoxin. Indeed, antitoxins from type II TA systems are known to be partially disordered proteins particularly upon dissociation from their cognate toxins [54], and an unstructured amino-terminus might be sufficient to target a protein for T3SA-mediated secretion [64]. Nevertheless, we initially identified by MS the endogenous products of *orf48* and *orf86* in *ipaD* supernatants, under conditions in which the natural toxin/antitoxin balance was not artificially disrupted, thus excluding an “in-absence-of-toxin” mechanism as an explanation for T3SA-mediated secretion of these antitoxins. To our knowledge, such secretion of antitoxins has not been previously reported. However, a recent study demonstrated antitoxin release through outer membrane vesicles in a Gram-negative plant pathogen [65], thereby shedding light on an alternative mechanism for disruption of the toxin-antitoxin balance beyond the canonical degradation by bacterial proteases.

What could be the role of antitoxins translocated by the T3SA? First, antitoxins could act as effectors. For example, their DNA binding capacity normally involved in modulating transcription of the TA systems could be harnessed to modulate host gene expression. Second, secretion of antitoxins could have a direct impact on bacterial fitness. We cannot totally discard the possibility that this secretion could induce bacterial killing [53,54], but given the observation that T3SA activity is transient [29], such a stark effect seems unlikely. One can therefore hypothesise that secretion of antitoxins belonging to functional TA systems such as Orf48 could momentarily slow down bacterial growth through the action of the freed toxin. Such a phenomenon might provide a protective mechanism to increase *Shigella* viability in the bactericidal vacuolar lumen [66], where ATG8/LC3 recruitment was indeed found to correlate with T3SA activity and effector translocation [67]. Interestingly, a *Salmonella enterica* Typhimurium toxin belonging to a TA module encoded on the bacterial chromosome and related to the GNAT superfamily of enzymes (e.g. *Shigella* Orf47 and Orf85) was recently shown to momentarily induce bacterial growth arrest under stress conditions [68]. These intracellular *Salmonella* persisters could later resume growth upon restoration of the antitoxin-toxin balance. In *Shigella*, T3SA-dependent secretion of antitoxins could momentarily decrease the bacterial division rate during the initial steps of invasion that rely on T3SA activity. Once in the host cell cytoplasm, where T3SA activity is switched off [29], *Shigella* would be able to increase its division rate back to a normal level, allowing efficient colonisation of the tissue. This hypothesis is consistent with data supporting secretion and translocation of the antitoxin Orf48, which is part of a functional TA system [53]. Effect of the secretion of the antitoxin of a non-functional TA system (Orf86) or of an orphan antitoxin (Orf176) on the bacterium and/or on its host is unclear. Overall, further work will be required to address the role of T3SA-mediated translocation of antitoxins.

The MS analysis presented in this study does not constitute a comprehensive screening of the *S*. *flexneri* T3SA-dependent secretome, as we focused on virulence plasmid-encoded proteins. Further work will be required to determine whether or not there are other chromosome-encoded proteins that are T3SA substrates beyond the chromosomal IpaHs. Nevertheless, using high-resolution MS systems, we were able to discover several additional T3SA substrates. Their molecular weight and their expression level are lower than known T3SA effectors (13±5 kDa versus 44±20 kDa and [48]). These characteristics might explain why they have escaped identification using traditional methods. Taken together, the results described herein illustrate how bacterial genetics, MS and secretion/translocation assays based on the β-lactamase or another relevant reporter protein can be combined to study the secretome of a bacterial pathogen. This strategy is in principle extendable to many other bacterial species expressing a T3SS or other contact mediated secretion systems (e.g. type IV and type VI secretion systems), provided that sufficient annotation of the genetic material is available and that relevant hyper-secreting and secretion-deficient strains can be obtained.

## Supporting information

S1 Table List of primers. (DOCX)

S2 Table List of plasmids. (DOCX)

S3 Table Mass spectrometry results. Quantification of peptide number and abundance in *ipaD* versus *mxiD* samples for the 3 biological replicates. High, intermediate and low confidence predictions are listed. Protein identification is given as GI accession number. NA: non applicable (one of the samples did not yield any peptide matching a protein hit).
(DOCX)

S1 Fig Study design. (TIF)

S2 Fig Expression of β-lactamase chimeric proteins initiated from different Shine and Dalgarno (SD) sequences. WT *Shigella* lysates were analysed by immunoblotting with anti-β-lactamase antibody. Load equivalent to a bacterial culture OD_600_ of 0.6 for each lane. cons: consensus SD; end: endogenous SD. Chimeric proteins tested and their expected molecular weight (kDa): Orf182-bla (140), OspI-bla (53), Orf86-bla (40), Orf48-bla (41), Orf176-bla (40), Orf13-bla (51), Orf131a-bla (38).
(TIF)

S3 Fig Assessment of β-lactamase assay reliability for measuring secretion by *ipaD* strains of substrates identified by MS. (**A**) Supernatants obtained after spinning of raw and sonicated sub-cultures (sub.) of *ipaD* strains expressing Orf182-bla, OspI-bla and Orf48-bla were incubated with nitrocefin. Enzymatic activity was calculated based on measurement of A_486_. e.M.u.: equivalent Miller unit. Data are from 3 independent experiments; *p<0.05; ***p<0.001 (unpaired two-tailed Student’s t-test) (**B-C**) *Shigella* lysates (pellet) and supernatants (sup.) from *ipaD* strains were analysed by immunoblotting. Load equivalent to a bacterial culture OD_600_ of 0.1 for each lane. Anti-IpaH antibody was used as a control for T3SA-mediated secretion (expected size 62 kDa). Data are representative of two independent experiments. (**B**) Immunoblotting with anti-c-Myc antibody assessing secretion of Orf182-myc (182, expected size 112 kDa) and OspI-myc (I, expected size 25 kDa) into *ipaD* supernatants. (**C**) Immunoblotting with anti-RecA antibody assessing release of cytosolic content into culture supernatants by the *ipaD* strain (expected size 38 kDa, runs at 42 kDa). *ipaD* strain devoid of Orf-bla (-) was used as a control. (**D**) *Shigella* lysates from Orf-bla-expressing *mxiD* strains were analysed by immunoblotting with anti-β-lactamase antibody. Load equivalent to a bacterial culture OD_600_ of 0.1 for each lane. See legend of Fig 3A for a description of their expected molecular weights. (**E**) Supernatants of *ipaD* and *mxiD* strains expressing the secreted Orf-bla chimeric proteins were incubated with nitrocefin. Enzymatic activity was calculated based on measurement of A_486_. e.M.u.: equivalent Miller unit. Data are from 4 independent experiments; **p<0.01; ***p<0.001; ****p<0.0001 (unpaired two-tailed Student’s t-test comparing both strains for each Orf-bla). (**F**) *Shigella* lysates (pellet) and supernatants (sup.) from Orf-bla-expressing WT strains upon induction by Congo Red (CR) were analysed by immunoblotting with anti-β-lactamase antibody. Load equivalent to a bacterial culture OD_600_ of 0.2 and 4 for pellets and supernatants, respectively.
(TIF)

S4 Fig Quantification of Orf-bla expression in bacterial lysates. Three independent sets of protein lysates from Orf-bla-expressing *ipaD* (**A**) and WT (**B**) *Shigella* were analysed by immunoblotting with anti-β-lactamase antibody. Intensity from the highest molecular weight species was quantified as a proportion of protein quantity measured in Orf182-bla-expressing bacteria. (1) Orf182-bla, (2) OspI-bla, (3) Orf86-bla, (4) Orf48-bla, (5) Orf176-bla, (6) Orf13-bla, (7) Orf131a-bla.
(TIF)

S5 Fig β-lactamase secretion assay control experiments for the WT *Shigella* strain used to assess translocation of substrates identified by MS. (**A**) WT *Shigella* lysates were analysed by immunoblotting with anti-β-lactamase antibody. Load equivalent to a bacterial culture OD_600_ of 0.2 for each condition. See legend of Fig 3A for expected sizes. (**B**) Supernatants of *ipaD* and WT strains expressing the Orf-bla chimeric proteins were incubated with nitrocefin. Enzymatic activity was calculated based on measurement of A_486_. e.M.u.: equivalent Miller unit. Data are from 3 independent experiments; **p<0.01; ***p<0.001; ****p<0.0001 (unpaired two-tailed Student’s t-test). (**C**) Flow cytometry data analysis: uninfected cells were used to define the gate selecting the targeted cells, identified among CCF2-AM-loaded Jurkat T cells as those with higher intensity values in the cleaved CCF2-AM fluorescence channel. (**D-E**) CCF2-AM-loaded Jurkat T lymphocytes were infected with WT or *mxiD* strains expressing Orf-bla chimeric proteins for 1 hour. Translocated cells were detected by flow cytometry. (**D**) Proportion of translocated cells detected. Data are from 3 independent experiments. **p<0.01; ***p<0.001; ****p<0.0001 (unpaired two-tailed Student’s t-test comparing both strains for each Orf-bla tested). (**E**) Representative flow cytometry dot plot of OspI-bla-infected cells. Black: CCF2-AM loaded cells; grey: non-loaded cells.
(TIF)

S6 Fig Plaque assay with single-mutant *Shigella* strains. Plaques resulting from invasion, intracellular replication and cell-to-cell spread within Caco-2/TC7 monolayers were enumerated (**A**) and their area measured (**B**) three days later. Data are from 4 independent experiments. No statistical difference between the different strains was found, as assessed by unpaired two-tailed Student’s t-test.
(TIF)

S7 Fig Assessment of β-lactamase assay reliability by measuring secretion and translocation of antitoxin and chaperones. (**A**) Lysates of WT *Shigella* expressing antitoxin and chaperone β-lactamase chimeric proteins were analysed by immunoblotting with anti-β-lactamase antibody. Load equivalent to a bacterial culture OD_600_ of 0.2 for each condition. See Fig 4A legend for expected sizes. (**B**) Supernatants of *ipaD* and WT strains expressing the antitoxin and chaperone β-lactamase chimeric proteins were incubated with nitrocefin. Enzymatic activity was calculated based on measurement of A_486_. e.M.u.: equivalent Miller unit. Data are from 3 independent experiments; *p<0.05; **p<0.01 (unpaired two-tailed Student’s t-test). (**C**) Supernatants obtained after spinning of raw and sonicated sub-cultures (sub.) of *ipaD* strains expressing Spa15-bla or IpgA-bla were incubated with nitrocefin. Enzymatic activity was calculated based on measurement of A_486_. e.M.u.: equivalent Miller unit. Data are from 3 independent experiments; ****p<0.0001 (unpaired two-tailed Student’s t-test). (**D-F**) *Shigella* lysates (pellet) and supernatants (sup.) from *ipaD* (**D**-**E**) and WT (**F**) strains were analysed by immunoblotting. Anti-IpaH antibody was used as a control to assess T3SA-mediated secretion (expected size 62 kDa). (**D**) Immunoblotting with anti-c-Myc antibody assessing secretion of Spa15-myc (15, expected size 16 kDa), IpgA-myc (A, expected size 16 kDa) and OspI-myc (I, expected size 25 kDa) into *ipaD* supernatants. (**E-F**) Immunoblotting with anti-Spa15 antibody assessing secretion of endogenous Spa15 (expected size 15 kDa) into *ipaD* (**E**) and WT (**F**) supernatants. Load equivalent to a bacterial culture OD_600_ of 0.1 (**D**-**E**) or 0.2 for pellet and 4 for supernatants (**F**) for each lane. (**G**) Supernatants of *ipaD* and *mxiD* strains expressing the antitoxin β-lactamase chimeric proteins were incubated with nitrocefin. Enzymatic activity was calculated based on measurement of A_486_. e.M.u.: equivalent Miller unit. Data are from 4 independent experiments; ***p<0.001 (unpaired two-tailed Student’s t-test).
(TIF)
