# Supplementary material for: Identification of novel substrates of Shigella T3SA through analysis of its virulence plasmid-encoded secretome
Source: PLoS One. 2017 Oct 26;12(10):e0186920. doi: 10.1371/journal.pone.0186920 (PMC5658099; doi:10.1371/journal.pone.0186920)
Supplement: S1 Table — (DOCX) [file pone.0186920.s001.docx]

**S1 Table. List of primers.**

Underlined sequences represent restriction enzyme sites used for cloning steps.

*Italics sequences* depict the “consensus” or “endogenous” SD sequence.

| Primers used to construct pSU2.1tt-bla (the bold segment depicts the sequence coding for the six amino acid linker upstream of the sequence coding for the mature TEM-1 β-lactamase): | | | |
| --- | --- | --- | --- |
| *tem1*_F | 5’-AGAGAGGGTACC*CTAGCTAGGAGAAATTAACC*ATG**CCCGGGGGTGGCGGA TCC**CACCCAGAAACGCTGG-3’ | | |
| *tem1*_R | 5’-AGAGAGTCTAGAGATTAATTACCAATGCTTAATCAGTGAGGC-3’ | | |
| Primers used to construct pSU2.1tt-Orf-bla plasmids: | | | |
| *ospI*_F | | 5’-AGAGAGGAATTC*CTAGCTAGGAGAAATTAACC*ATGATTAATGGGGTGTC GTTACAG -3’ | |
| *ospI*_R | | 5’-AGAGAGCCCGGGGCAAAGCCTCTTACTTTTCCAACTACTA -3’ | |
| *orf86*_F | | 5’-AGAGAGGAATTC*CTAGCTAGGAGAAATTAACC*ATGATATACGGAGGCTTT ATGAAATCA -3’ | |
| *orf86*_R | | 5’-AGAGAGCCCGGGCACATCCCATTGAGGTTTCCTT -3’ | |
| *orf48*_F | | 5’-AGAGAGGAATTC*CTAGCTAGGAGAAATTAACC*ATGGAGGTTTTTATGTCT ACAGCTG -3’ | |
| *orf48_*R | | 5’-AGAGAGCCCGGGTTTCCATTCAGGCTTTACCG-3’ | |
| *orf176*_F | | 5’-AGAGAGGAATTC*CTAGCTAGGAGAAATTAACC*ATGGCACAGGTTAATAT GAGTGTAAGAAT-3’ | |
| *orf176*_R | | 5’-AGAGAGCCCGGGTTTATCAGCAATCTTCCTGCTCATT-3’ | |
| *orf13*_F | | 5’-AGAGAGGAATTC*CTAGCTAGGAGAAATTAACC*ATGAAAGTCTCGTTTAA GTCTTTAGGCTATA-3’ | |
| *orf13*_R | | 5’-AGAGAGCCCGGGAACACATTTATATAAGTTCTTTGCTGAAC AA-3’ | |
| *orf131a*_F | | 5’-AGAGAGGAATTC*CTAGCTAGGAGAAATTAACC*ATGTGTTATATGGGAGT TAATTTCTGTAATAAAA-3’ | |
| *orf131a*_R | | 5’-AGAGAGCCCGGGTAAATCATCAGGTGTTTTTTCAACG-3’ | |
| *orf182*_F | | 5’-AGAGAGGAATTC*CTAGCTAGGAGAAATTAACC*TTGTTCAGTAAGGCATTC CTTCG-3’ | |
| *orf182*_R | | 5’-AGAGAGCAGCTGCGCTTTTTGAGCGGCAC-3’ | |
| Primers used to construct pSU2.1tt-endSD-Orf-bla plasmids: | | | |
| *endSDorf13_F* | | | 5’-AGAGAGGAATTC*GATATATATATATCCTGTTAATATTTAA*-3’ |
| *endSDorf131a_F* | | | 5’-*CTCATTAATAAGTAAAGGAG*ATGTGTTATATGGGAGTTAATTTCTG TAATAAAA-3’ |
| *endSDorf131a_R* | | | 5’-*TGTTTTCAACCTGC*TCAAGCGAATTCGTAATCTTAGCTAGTTACTC GAG-3’ |
| *endSDorf182_F* | | | 5’-*GGGGGGTATTCCGGGCAAGG*TTGTTCAGTAAGGCATTC CTTCG-3’ |
| *endSDorf182_R* | | | 5’-*GAAATCAGATGGAACAATCA*GAATTCGTAATCTTAGCTAGTTACTC GAG-3’ |
| *endSDorf176_F* | | | 5’-*TGGTAATGACGAGGTGGCAA*ATGGCACAGGTTAATATGAGTGTA AG-3’ |
| *endSDorf176_R* | | | 5’-*CGTATATACACAGTATATAA*GAATTCGTAATCTTAGCTAGTTACTCG AG-3’ |
| *endSDospI_F* | | | 5’-*ATATACTGTCAGGATACAAT*ATGATTAATGGGGTGTCGTT ACA-3’ |
| *endSDospI_R* | | | 5’-*ATACTCACTATATTTTCTTC*GAATTCGTAATCTTAGCTAGTTACTCG AG-3’ |
| *endSDorf86_F* | | | 5’-*ATGCTATTGTTGTAAAGCAA*ATGATATACGGAGGCTTTAT GAAA TC-3’ |
| *endSDorf86_R* | | | 5’-*ATTGACAATATATAGCAAAT*GAATTCGTAATCTTAGCTAGTTACTCG AG-3’ |
| *endSDorf48_F* | | | 5’-*TGCATAGCAAATTGTTACCA*ATGGAGGTTTTTATGTCTAC AGCTG-3’ |
| *endSDorf48_R* | | | 5’-*ATTAATGTTATCGCAAATGG*GAATTCGTAATCTTAGCTAGTTACTCG  AG-3’ |
| *endSDmvpA_F* | | | 5’-AGAGAGGAATTC*ATAGATATACACAAGACATATCCACAT*-3’ |
| *endSDmvpA_R* | | | 5’-AGAGAGCCCGGGGAATGACTCCCTTTCCTG-3’ |
| *endSDccdA_F* | | | 5’-AGAGAGGAATTC*ACTCATATACATATCATAAGTATGTTTTTG*-3’ |
| *endSDccdA_R* | | | 5’-AGAGAGCCCGGGCCAGTCCCTGTTCTCATC-3’ |
| *endSDspa15_F* | | | 5’-AGAGAGGAATTC*TACCATTAATGTATTAAAGACTATTTAG*-3’ |
| *endSDspa15_R* | | | 5’-AGAGAGCCCGGGTAAGACCCCATTTAAGATTTC-3’ |
| *endSDipgA_F* | | | 5’-AGAGAGGAATTC*ATTGAATAACTCTCATTCTAATATATAG*-3’ |
| *endSDipgA_R* | | | 5’-AGAGAGCCCGGGGTTCACTTCTGAAGTGATGTTT-3’ |
| Primers used to construct pSU2.1tt-endSD-Orf-myc plasmids (the bold segment depicts the sequence coding for the c-Myc tag): | | | |
| *Bla_to_myc_F* | | | 5’-**ATTAGCGAAGAGGATCT**GTAATTAATCTCTAGAGTCGACCTG-3’ |
| *Orf182myc_R* | | | 5’-**AAGCTTCTGTTCTCCGGA**CGCTTTTTGAGCGGC-3’ |
| *OspImyc_R* | | | 5’-**AAGCTTCTGTTCTCCGGA**GCAAAGCCTCTTACTTTTCC-3’ |
| *Spa15myc_R* | | | 5’-**AAGCTTCTGTTCTCCGGA**TAAGACCCCATTTAAGATTTC-3’ |
| *IpgAmyc_R* | | | 5’-**AAGCTTCTGTTCTCCGGA**GTTCACTTCTGAAGTGATGTTT-3’ |
