# Supplementary material for: Identification of novel substrates of Shigella T3SA through analysis of its virulence plasmid-encoded secretome
Source: PLoS One. 2017 Oct 26;12(10):e0186920. doi: 10.1371/journal.pone.0186920 (PMC5658099; doi:10.1371/journal.pone.0186920)
Supplement: S2 Table — (DOCX) [file pone.0186920.s002.docx]

**S2 Table. List of plasmids.**

| **Name** | **Description** |
| --- | --- |
| pSU2.1tt | pSU2718 with modification of the multiple cloning site and insertion of the transcription terminator of *trpA* upstream of the lac promoter |
| pSU2.1tt-bla | pSU2.1tt with insertion downstream of the lac promoter of a “consensus” SD sequence, a six amino acids linker and the sequence coding for the mature β-lactamase TEM-1 extracted from pUC18 |
| pSU2.1tt-Orf-bla | pSU2.1tt-bla with insertion of the sequence coding for pWR100-encoded ORFs of interest upstream and in frame with the sequences coding for the six amino acids linker and the mature β-lactamase TEM-1 |
| pSU2.1tt-endSD-Orf-bla | pSU2.1tt-Orf-bla with exchange of the “consensus” SD for the “endogenous” SD (endSD) corresponding to the 30 to 40 nucleotides upstream of the start codon of the corresponding ORF in pWR100 sequence |
| pSU2.1tt-endSD-Orf-myc | pSU2.1tt-endSD-Orf-bla with exchange of the β-lactamase coding sequence for the smaller c-Myc tag coding sequence |
| pCP20 | Plasmid expressing the flippase recombinase targeting FRT sites [44] |
| pUC18ΔZ-DsRed | pUC18ΔZ-TSAR (pFX4 plasmid [29] with zeocyn resistance instead of ampicillin) with the insert replaced by the sequence coding for the DsRed under the control of the *rpsM* promoter from pTSAR1.3 [29]. |
