# Supplementary material for: Identification of novel substrates of Shigella T3SA through analysis of its virulence plasmid-encoded secretome
Source: PLoS One. 2017 Oct 26;12(10):e0186920. doi: 10.1371/journal.pone.0186920 (PMC5658099; doi:10.1371/journal.pone.0186920)
Supplement: S3 Table — Quantification of peptide number and abundance in ipaD versus mxiD samples for the 3 biological replicates. High, intermediate and low confidence predictions are listed. Protein identification is given as GI accession number. NA: non applicable (one of the samples did not yield any peptide matching a protein hit). (DOCX) [file pone.0186920.s003.docx]

**S3 Table. Mass spectrometry results**
