# Supplementary figures and images for: Identification of novel substrates of Shigella T3SA through analysis of its virulence plasmid-encoded secretome
Source: PLoS One. 2017 Oct 26;12(10):e0186920. doi: 10.1371/journal.pone.0186920 (PMC5658099; doi:10.1371/journal.pone.0186920)

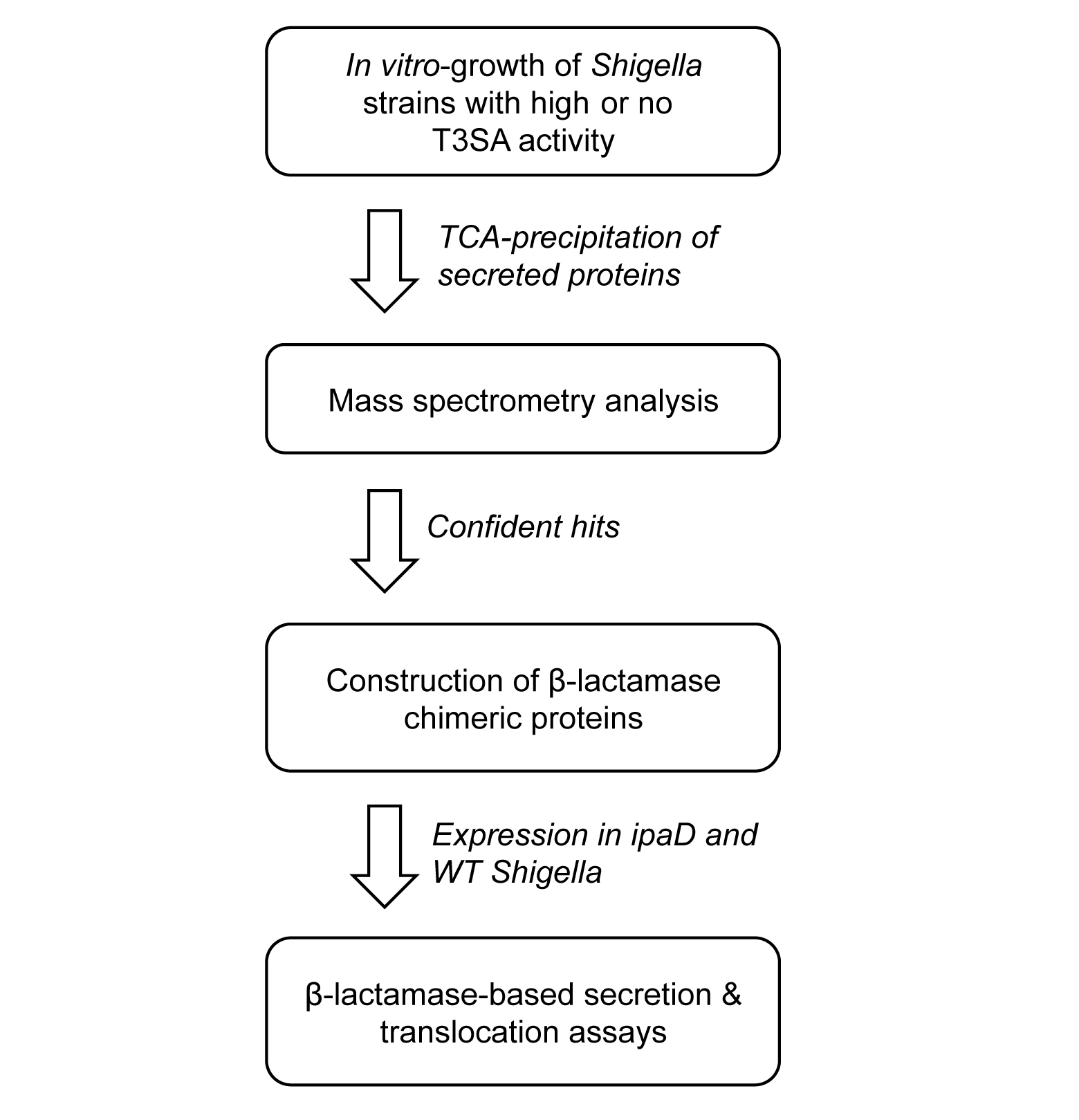

Supplement: S1 Fig — (TIF) [file pone.0186920.s004.tif]

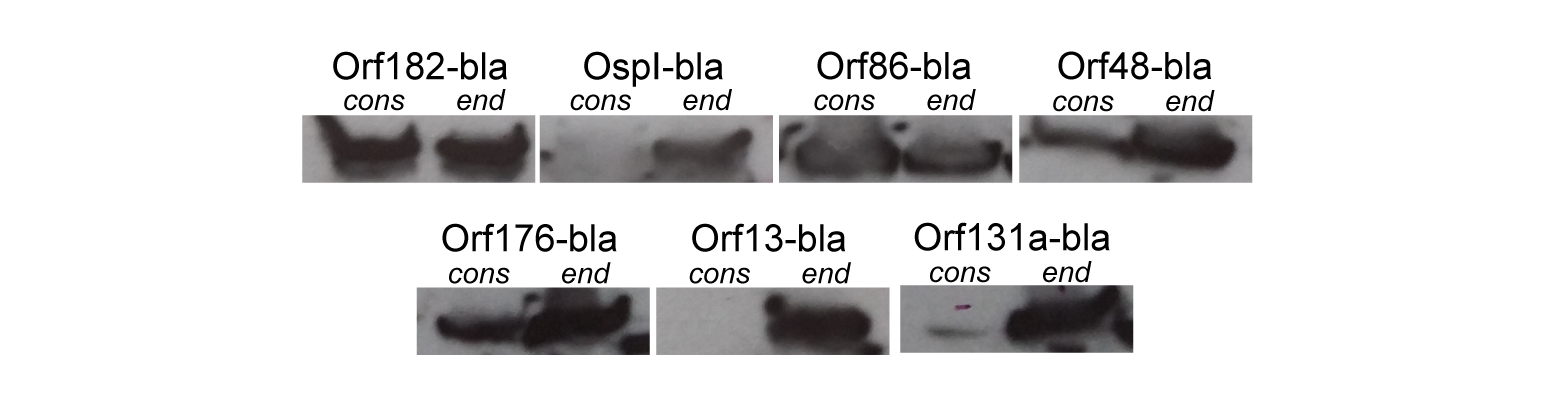

Supplement: S2 Fig — WT Shigella lysates were analysed by immunoblotting with anti-β-lactamase antibody. Load equivalent to a bacterial culture OD600 of 0.6 for each lane. cons: consensus SD; end: endogenous SD. Chimeric proteins tested and their expected molecular weight (kDa): Orf182-bla (140), OspI-bla (53), Orf86-bla (40), Orf48-bla (41), Orf176-bla (40), Orf13-bla (51), Orf131a-bla (38). (TIF) [file pone.0186920.s005.tif]

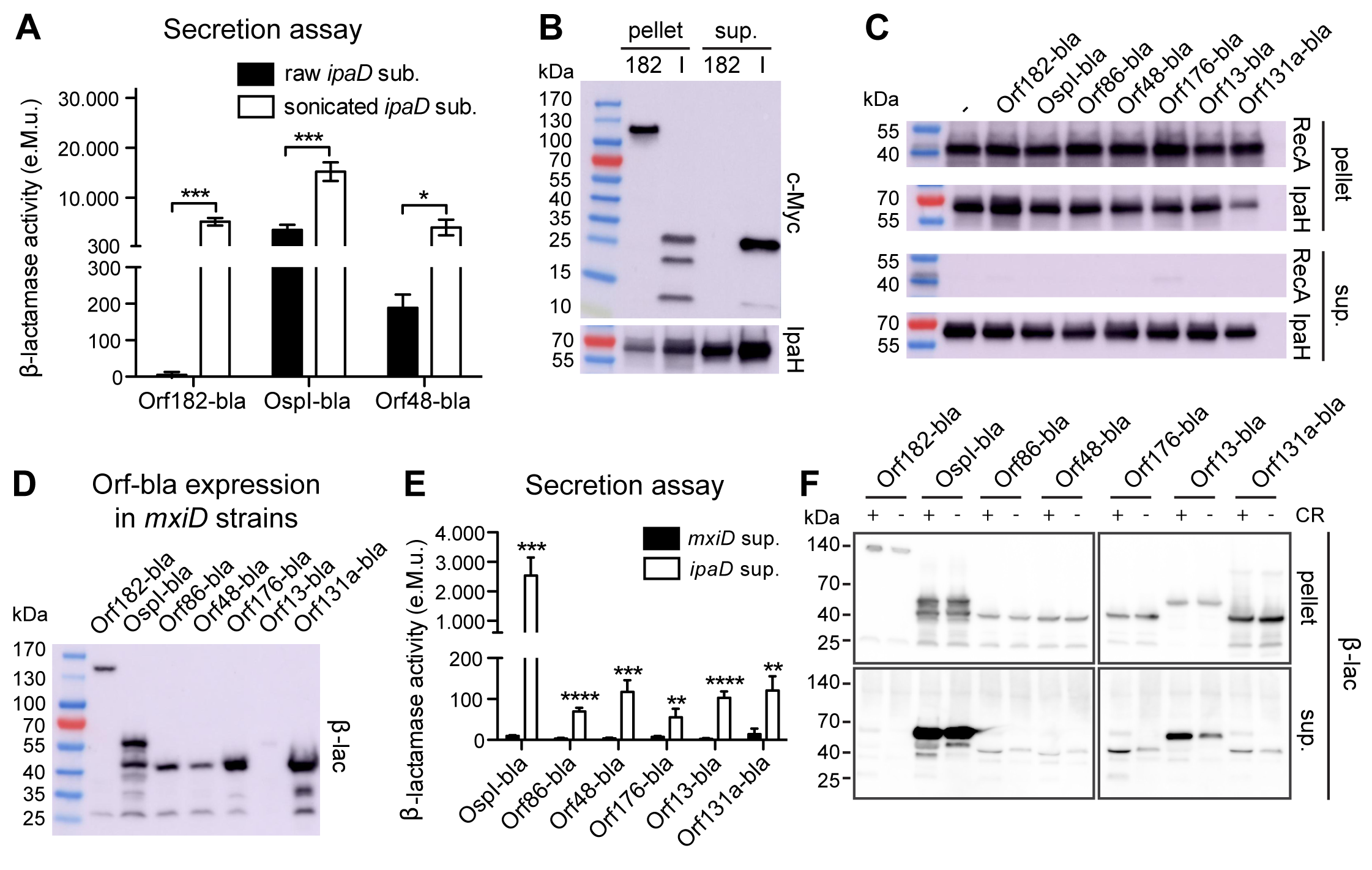

Supplement: S3 Fig — (A) Supernatants obtained after spinning of raw and sonicated sub-cultures (sub.) of ipaD strains expressing Orf182-bla, OspI-bla and Orf48-bla were incubated with nitrocefin. Enzymatic activity was calculated based on measurement of A486. e.M.u.: equivalent Miller unit. Data are from 3 independent experiments; *p<0.05; ***p<0.001 (unpaired two-tailed Student’s t-test) (B-C) Shigella lysates (pellet) and supernatants (sup.) from ipaD strains were analysed by immunoblotting. Load equivalent to a bacterial culture OD600 of 0.1 for each lane. Anti-IpaH antibody was used as a control for T3SA-mediated secretion (expected size 62 kDa). Data are representative of two independent experiments. (B) Immunoblotting with anti-c-Myc antibody assessing secretion of Orf182-myc (182, expected size 112 kDa) and OspI-myc (I, expected size 25 kDa) into ipaD supernatants. (C) Immunoblotting with anti-RecA antibody assessing release of cytosolic content into culture supernatants by the ipaD strain (expected size 38 kDa, runs at 42 kDa). ipaD strain devoid of Orf-bla (-) was used as a control. (D) Shigella lysates from Orf-bla-expressing mxiD strains were analysed by immunoblotting with anti-β-lactamase antibody. Load equivalent to a bacterial culture OD600 of 0.1 for each lane. See legend of Fig 3A for a description of their expected molecular weights. (E) Supernatants of ipaD and mxiD strains expressing the secreted Orf-bla chimeric proteins were incubated with nitrocefin. Enzymatic activity was calculated based on measurement of A486. e.M.u.: equivalent Miller unit. Data are from 4 independent experiments; **p<0.01; ***p<0.001; ****p<0.0001 (unpaired two-tailed Student’s t-test comparing both strains for each Orf-bla). (F) Shigella lysates (pellet) and supernatants (sup.) from Orf-bla-expressing WT strains upon induction by Congo Red (CR) were analysed by immunoblotting with anti-β-lactamase antibody. Load equivalent to a bacterial culture OD600 of 0.2 and 4 for pellets and supernatan [file pone.0186920.s006.tif]

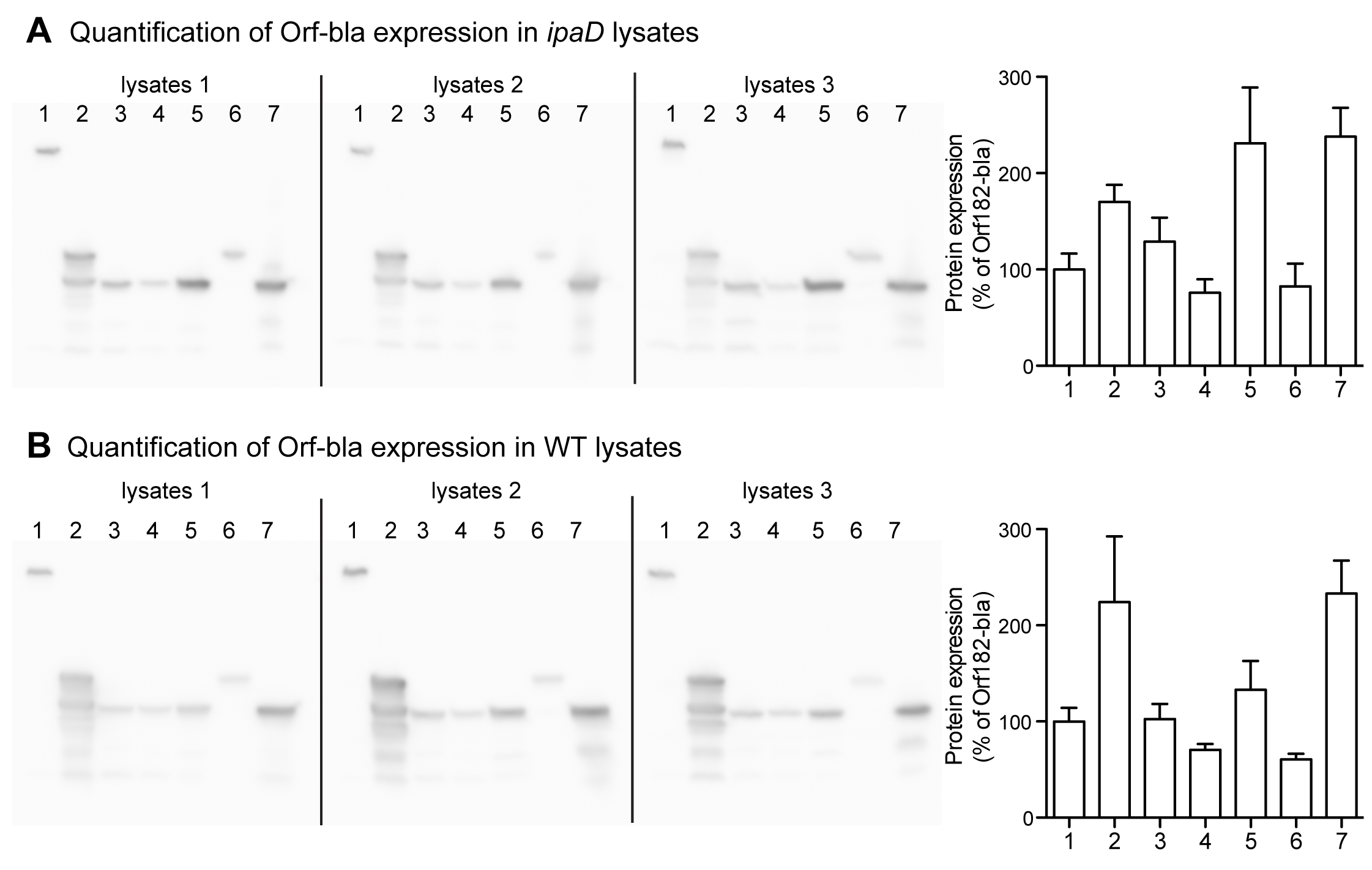

Supplement: S4 Fig — Three independent sets of protein lysates from Orf-bla-expressing ipaD (A) and WT (B) Shigella were analysed by immunoblotting with anti-β-lactamase antibody. Intensity from the highest molecular weight species was quantified as a proportion of protein quantity measured in Orf182-bla-expressing bacteria. (1) Orf182-bla, (2) OspI-bla, (3) Orf86-bla, (4) Orf48-bla, (5) Orf176-bla, (6) Orf13-bla, (7) Orf131a-bla. (TIF) [file pone.0186920.s007.tif]

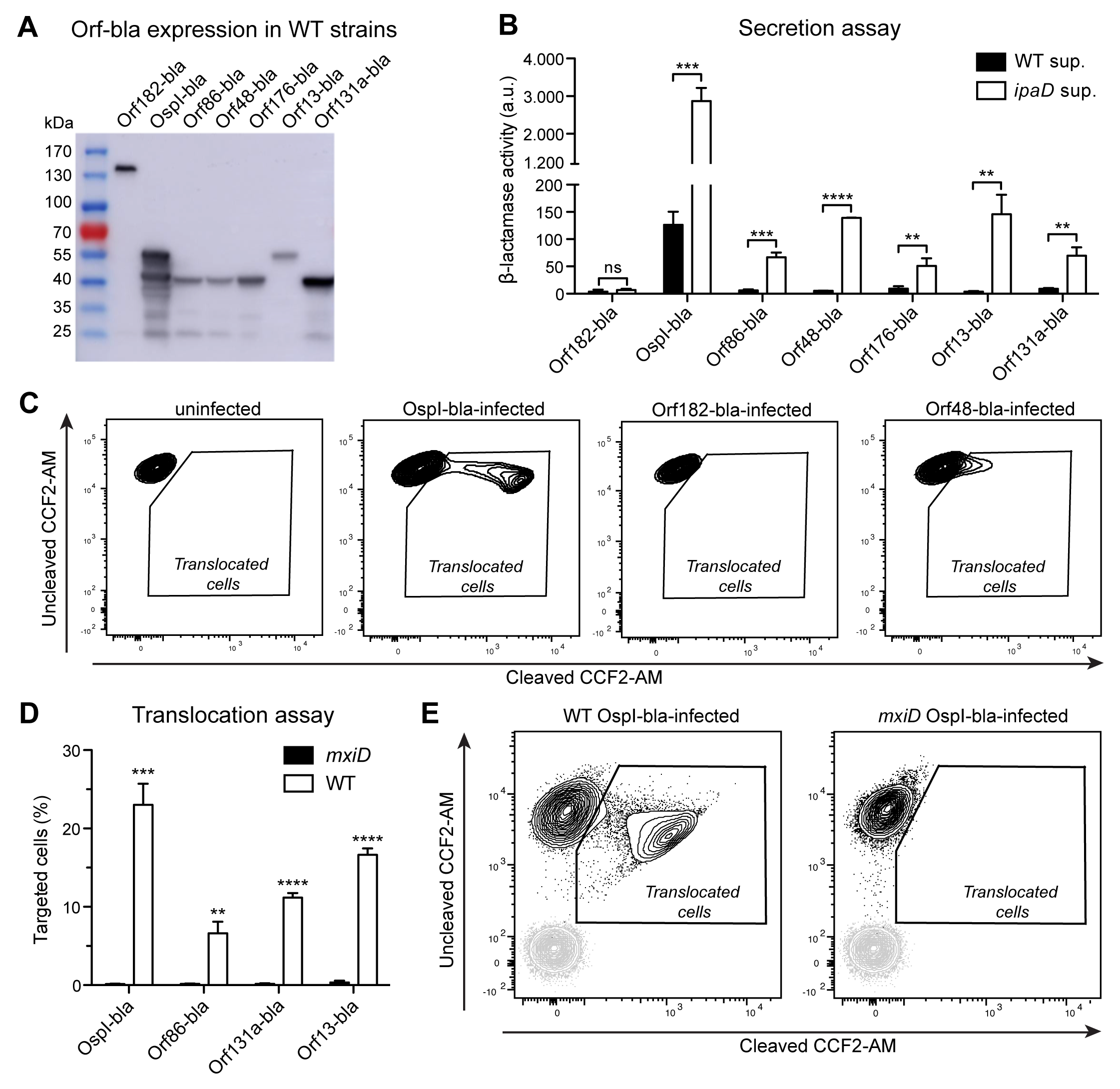

Supplement: S5 Fig — (A) WT Shigella lysates were analysed by immunoblotting with anti-β-lactamase antibody. Load equivalent to a bacterial culture OD600 of 0.2 for each condition. See legend of Fig 3A for expected sizes. (B) Supernatants of ipaD and WT strains expressing the Orf-bla chimeric proteins were incubated with nitrocefin. Enzymatic activity was calculated based on measurement of A486. e.M.u.: equivalent Miller unit. Data are from 3 independent experiments; **p<0.01; ***p<0.001; ****p<0.0001 (unpaired two-tailed Student’s t-test). (C) Flow cytometry data analysis: uninfected cells were used to define the gate selecting the targeted cells, identified among CCF2-AM-loaded Jurkat T cells as those with higher intensity values in the cleaved CCF2-AM fluorescence channel. (D-E) CCF2-AM-loaded Jurkat T lymphocytes were infected with WT or mxiD strains expressing Orf-bla chimeric proteins for 1 hour. Translocated cells were detected by flow cytometry. (D) Proportion of translocated cells detected. Data are from 3 independent experiments. **p<0.01; ***p<0.001; ****p<0.0001 (unpaired two-tailed Student’s t-test comparing both strains for each Orf-bla tested). (E) Representative flow cytometry dot plot of OspI-bla-infected cells. Black: CCF2-AM loaded cells; grey: non-loaded cells. (TIF) [file pone.0186920.s008.tif]

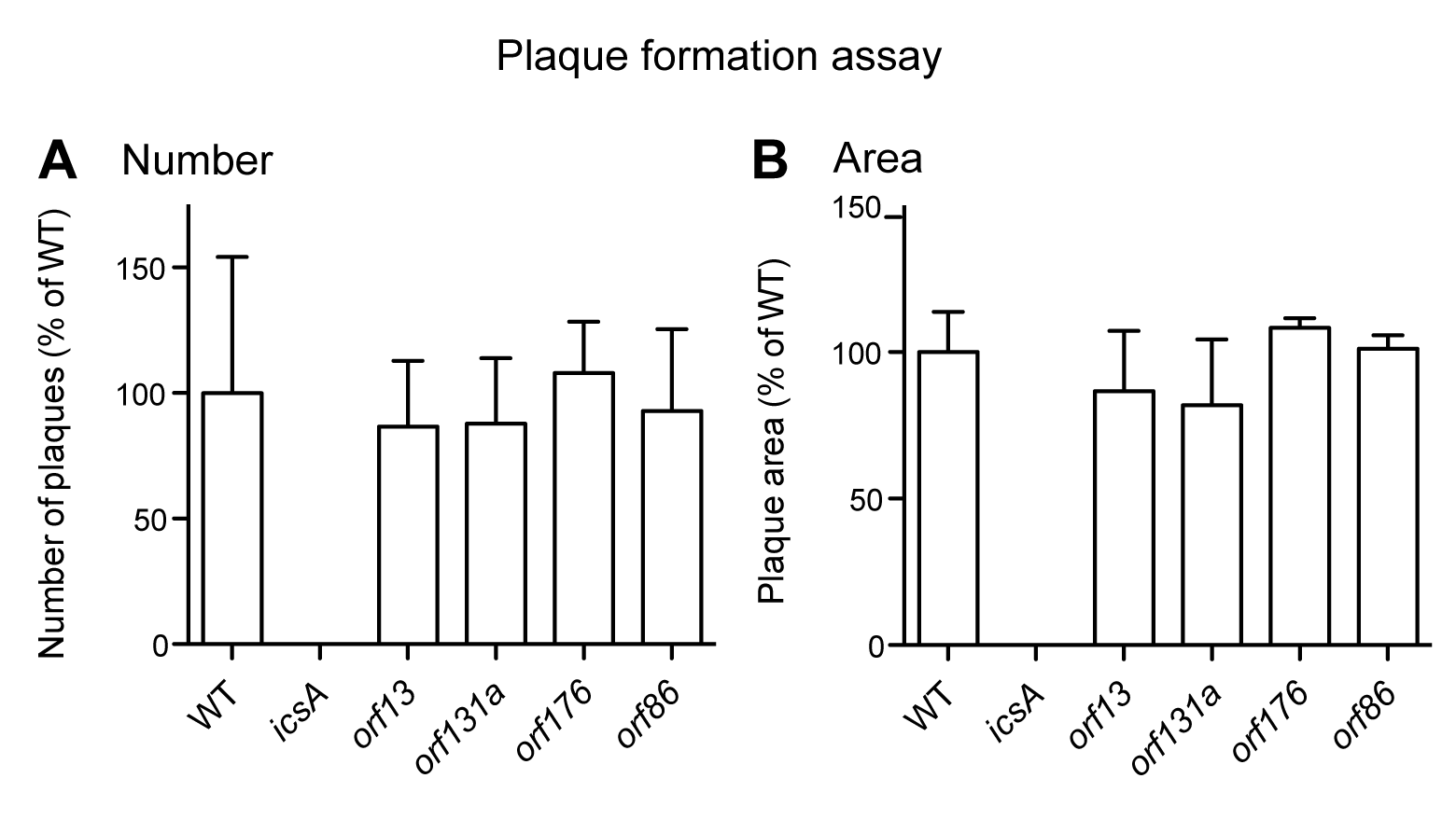

Supplement: S6 Fig — Plaques resulting from invasion, intracellular replication and cell-to-cell spread within Caco-2/TC7 monolayers were enumerated (A) and their area measured (B) three days later. Data are from 4 independent experiments. No statistical difference between the different strains was found, as assessed by unpaired two-tailed Student’s t-test. (TIF) [file pone.0186920.s009.tif]

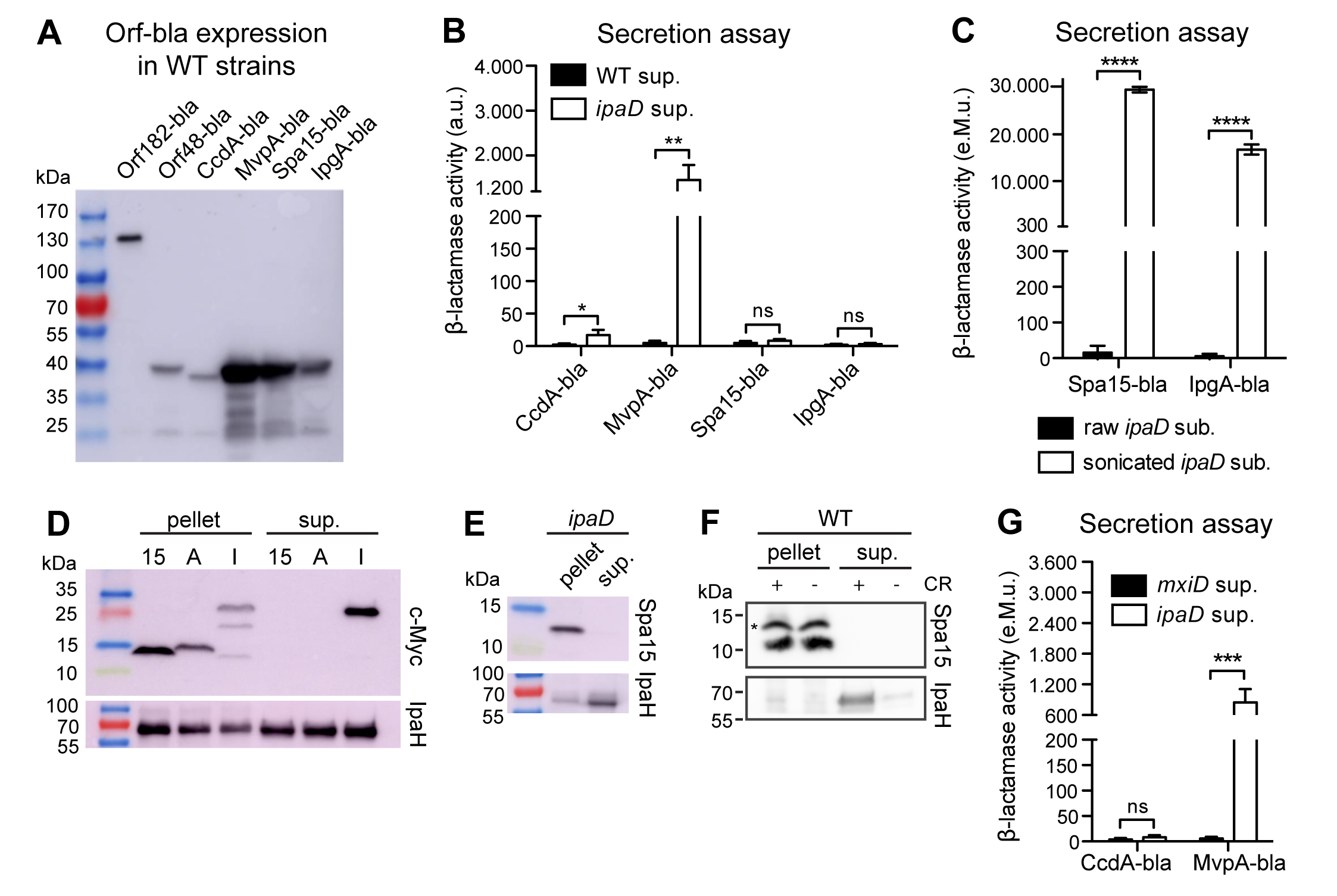

Supplement: S7 Fig — (A) Lysates of WT Shigella expressing antitoxin and chaperone β-lactamase chimeric proteins were analysed by immunoblotting with anti-β-lactamase antibody. Load equivalent to a bacterial culture OD600 of 0.2 for each condition. See Fig 4A legend for expected sizes. (B) Supernatants of ipaD and WT strains expressing the antitoxin and chaperone β-lactamase chimeric proteins were incubated with nitrocefin. Enzymatic activity was calculated based on measurement of A486. e.M.u.: equivalent Miller unit. Data are from 3 independent experiments; *p<0.05; **p<0.01 (unpaired two-tailed Student’s t-test). (C) Supernatants obtained after spinning of raw and sonicated sub-cultures (sub.) of ipaD strains expressing Spa15-bla or IpgA-bla were incubated with nitrocefin. Enzymatic activity was calculated based on measurement of A486. e.M.u.: equivalent Miller unit. Data are from 3 independent experiments; ****p<0.0001 (unpaired two-tailed Student’s t-test). (D-F) Shigella lysates (pellet) and supernatants (sup.) from ipaD (D-E) and WT (F) strains were analysed by immunoblotting. Anti-IpaH antibody was used as a control to assess T3SA-mediated secretion (expected size 62 kDa). (D) Immunoblotting with anti-c-Myc antibody assessing secretion of Spa15-myc (15, expected size 16 kDa), IpgA-myc (A, expected size 16 kDa) and OspI-myc (I, expected size 25 kDa) into ipaD supernatants. (E-F) Immunoblotting with anti-Spa15 antibody assessing secretion of endogenous Spa15 (expected size 15 kDa) into ipaD (E) and WT (F) supernatants. Load equivalent to a bacterial culture OD600 of 0.1 (D-E) or 0.2 for pellet and 4 for supernatants (F) for each lane. (G) Supernatants of ipaD and mxiD strains expressing the antitoxin β-lactamase chimeric proteins were incubated with nitrocefin. Enzymatic activity was calculated based on measurement of A486. e.M.u.: equivalent Miller unit. Data are from 4 independent experiments; ***p<0.001 (unpaired two-tailed Student’s t-test). (TIF) [file pone.0186920.s010.tif]
